# Comparison of inflammatory biomarker levels in neurodegenerative proteinopathies: a case-control study

**DOI:** 10.1007/s00702-025-02902-6

**Published:** 2025-03-03

**Authors:** Sarah E. V. Cook, Kateřina Menšíková, Dorota Koníčková, Hedvika Šlanhofová, Kateřina Klíčová, Milan Raška, Jana Zapletalová, David Friedecký, Petr Kaňovský

**Affiliations:** 1 Department of Neurology, Faculty of Medicine and Dentistry, Palacký University, Olomouc, Czech Republic; 2 Department of Neurology, University Hospital Olomouc, Olomouc, Czech Republic; 3 Department of Immunology, University Hospital Olomouc, Olomouc, Czech Republic; 4 Department of Immunology, Faculty of Medicine and Dentistry, Palacký University, Olomouc, Czech Republic; 5 Department of Medical Biophysics, Faculty of Medicine and Dentistry, Palacký University, Olomouc, Czech Republic; 6 Department of Clinical Biochemistry, University Hospital Olomouc, Olomouc, Czech Republic; 7 Laboratory for Inherited Metabolic Disorders, Faculty of Medicine and Dentistry, Palacký University, Olomouc, Czech Republic

**Keywords:** Neuroinflammation, Biomarkers, Alpha-synucleinopathies, 4-repeat tauopathies, Parkinsonism, Neurodegenerative diseases

## Abstract

While diagnostic criteria have been established and validated for most neurodegenerative diseases, the considerable overlap between individual nosological entities remains a significant diagnostic challenge. Increasing evidence suggests that neurodegeneration is often initiated by inflammation within the central nervous system. The identification of inflammation could serve as a first signal of the pathophysiological process. As such, validated biological markers (“biomarkers”) of neuroinflammation are critically important. This study aimed to assess the presence and levels of inflammatory biomarkers in three neurodegenerative diseases: Lewy body diseases (LBD), multiple system atrophy (MSA), and 4-repeat tauopathies (4RT). A total of 83 LBD, 24 MSA, and 31 4RT patients were included, with 83 control subjects for comparison. Six immune-related proteins were analysed in cerebrospinal fluid (CSF) and blood serum (serum): C3 complement, C4 complement, haptoglobin, transferrin, orosomucoid, and β2 microglobulin (β2M). ANCOVA statistical analysis revealed significantly lower levels of several inflammatory biomarkers in LBD (CSF: transferrin, C3 complement, orosomucoid; Serum: orosomucoid, β2M) and MSA (CSF: transferrin, C3 complement, C4 complement, orosomucoid) compared to controls. Significant differences were also observed between the synucleinopathy patient groups (LBD and MSA) and 4RT in serum levels of C3 complement. Additionally, the CSF/serum quotients for transferrin (LBD and MSA) and C3 complement (LBD) were significantly lower in disease relative to controls. These findings suggest that inflammatory processes may play a role in the pathophysiology of neurodegenerative proteinopathies, warranting further research to confirm these associations. The identification of potential fluid biomarkers would then represent a promising step forward in the field.

**Supplementary Information:**

The online version contains supplementary material available at 10.1007/s00702-025-02902-6.

## Introduction

The role of neuroinflammation in the pathophysiology of neurodegeneration has become a central focus of research in recent years, with ever increasing evidence that neuroinflammation may be a key factor in the development and/or progression of neurodegeneration. However, several aspects remain contentious, particularly whether neuroinflammation is primarily protective or harmful, and at what stage the balance between these effects is disrupted (Kwon and Koh 2020; Mishra et al. 2021; Balistreri and Monastero 2023; Zhang et al. 2023; Adamu et al. 2024; Giri et al. 2024). The term “neuroinflammation” refers to a repertoire of responses which are triggered when damage or disease is detected within the central nervous system (CNS). This leads to a typical inflammatory response, with the activation of complement, release of cytokines and chemokines, and the activation of the local immune cells, including microglia and astrocytes (Surendranathan et al. 2015). Additionally, circulating immune cells can infiltrate the CNS (Surendranathan et al. 2015), and immune dysregulation has been reported in both the periphery and brain of patients with Parkinson’s disease (PD). Elevated levels of pro-inflammatory cytokines have been detected in the blood serum (serum) of PD patients, suggesting that these chemical mediators may be associated with the onset and development of neuroinflammation (Reale et al. 2009; Wang et al. 2021; Tansey et al. 2022).

As reviewed by Koníčková et al. (2022), the majority of biomarkers in neurodegenerative disease research are derived from the pathological proteins characteristic of each disease, the proteins involved in their production, proteins indicative of neuronal degradation, and/or proteins indicative of synaptic dysfunction (Konícková et al. 2022). Other studies, such as the meta-analysis by Qu et al. (2023), have investigated classic inflammatory cytokines and chemokines as biomarkers (Qu et al. 2023). Current research around synucleinopathies and tauopathies focus more on imaging techniques or the different forms of tau protein and amyloid-beta, cytokines, or neurofilament light chain in the serum or CSF (Saeed et al. 2017; Hall et al. 2018; Starhof et al. 2018; Santaella et al. 2020; Chouliaras et al. 2022; Tokutake et al. 2022), so there are a lack of publications investigating other biomarkers. While many potential biomarkers have been identified, few have been validated. Therefore, the gaps in biomarker research remain until reliable biomarkers are confirmed.

The present study centres on six specific inflammatory biomarkers from the acute phase proteins group, all of which are available for routine examination in the majority of hospitals worldwide - transferrin, orosomucoid, haptoglobin, C3 complement, C4 complement, and β2M. Both “positive” and “negative” acute phase responding representatives were selected, including those which are preferentially produced in the liver (transferrin, orosomucoid, haptoglobin, C3 complement, C4 complement), of which C3, C4, and orosomucoid are also reported to be produced in activated brain astrocytes, and β2M as the representative without preferential liver production. During the selection, the involvement of those with well-known proinflammatory (C3, C4) and immunomodulatory (orosomucoid) functions were also considered.

The proteins of the complement system are mainly produced in the liver but several reports also detected C3 production in microglia-activated astrocytes (Chi et al. 2025). The complement system is involved in the development and homeostasis of neural networks but increased activation of the complement in the substantia nigra is likely damaging (Loeffler et al. 2006; Gregersen et al. 2021). Serum complement C3 and C4 are accepted as inflammatory biomarkers for many disorders, but under conditions with modest systemic activation, local complement consumption could lead to a reduction in its normal levels. The study by Gregersen et al. (2021) found that α-syn-dependent complement activation in neurons increases C3b deposition, which may contribute to microglial activation, while C4b deposition can also occur from activation of the classical complement pathway via immobilized α-syn (Gregersen et al. 2021). Other studies found immunohistochemical positivity for complement factors C3d/C3b and C4d in Lewy bodies in the substantia nigra (Yamada et al. 1992; Loeffler et al. 2006), and an increase in C3 complement in AD and animal models (Wu et al. 2019; Bourel et al. 2021). While research exists on C3 and C4 complement in neurodegeneration, much of it relates to Alzheimer’s disease (AD) or animal models, and so the present study wished to observe their concentrations within the parkinsonian disorders cohort of the study.

Human transferrin (Tf) is mainly produced in the liver, is an iron-binding protein, and its concentration decreases in association with hepatic dysfunction or inflammation (Kawabata 2019). Involvement of Tf in PD was studied by Ayton et al. (2016), who reported that the tissue concentration of Tf is decreased in the substantia nigra of PD patients (Ayton et al. 2016), possibly due to Tf trafficking of iron away from the tissue, mis-localisation of Tf (Gille and Reichmann 2011; Ayton et al. 2016), or decreased production of Tf (Pfeiffer and Looker 2017; Dignass et al. 2018). However, studies involving Tf in parkinsonism and neurodegeneration are lacking.

Orosomucoid is an acute phase protein synthesised mainly in the liver, lymphocytes and epithelial cells, with possible expression of orosomucoid by reactive astrocytes (Jo et al. 2017). Orosomucoid appears to play some role in immune regulation and inflammation responses, with its glycosylation pattern determining its effects (Ceciliani and Lecchi 2019; Elpek 2021). In the brain, orosomucoid is proposed to act as a mediator of astrocyte-microglial interaction, and particularly orosomucoid 2 exerts anti-inflammatory effects by modulating microglial activation and migration during brain inflammation (Jo et al. 2017). One study found little to no changes between PD patients and controls (Dufek et al. 2009), while another recent study discovered downregulation in the PD amygdala (Villar-Conde et al. 2023). However, the biological role of orosomucoid is not fully elucidated and little is known about the involvement of orosomucoid in AD and PD.

Haptoglobin is mainly synthesised in the liver (Wang et al. 2001) but is present within the brain at very low concentrations under normal conditions (Zhao et al. 2009; Garland et al. 2020). It is a haemoglobin binding protein that protects against oxidative stress by removing free haemoglobin from tissues and the circulation. It is also important in immune system regulation, inflammation suppression, angiogenesis, and chaperone functions, with the majority of neurodegenerative disease studies focusing on, or related to, AD (Yerbury et al. 2005; Costa-Mallen et al. 2008; Cocciolo et al. 2012; Chaubey et al. 2016; Naryzny and Legina 2021; Philbert et al. 2021). Studies have found that the genotype of haptoglobin may influence the diagnosis, prognosis and/or progression of several neuroinflammatory/neurodegenerative diseases, including multiple sclerosis (Bamm et al. 2017) and PD (Costa-Mallen et al. 2008). However, there are few studies investigating this.

β2M is an essential part of the major histocompatibility class I complex presented on most nucleated cells (Qun et al. 2019) and serum level likely reflects the cell membrane revival and cell turnover rate. β2M increases with several infectious, autoimmune, and CNS diseases and infection/disease-associated inflammation (Yekani and Memar 2023). β2M may act as a chaperone to α-syn, and possibly other proteins, in that it prevents its aggregation, but does not disrupt preformed fibrils (Rani et al. 2023). Thus, decreased levels of β2M may be associated with LBD, where α-syn aggregation is a pathological characteristic. There has also been a recent discovery of an association of β2M with amyloid beta and amyloid plaques in AD (Zhao et al. 2023). Nevertheless, β2M is not well understood in neurodegeneration.

The brain can receive inflammatory input from the peripheral nervous system (PNS), as well as the CNS itself. There are two pathways by which pro-inflammatory cytokines can reach the brain from the periphery: (1) the traditional endocrine pathway, i.e., tight binding to endothelial cells of the blood-brain barrier (BBB), followed by the destruction of the BBB lining, allowing penetration; (2) by direct nerve transmission, e.g. via the afferent vagus nerve (Wang et al. 2021). The BBB may also be disrupted by pathological proteins. α-syn was found to decrease the viability of, and increase the production of reactive oxygen species in, the cells associated with the BBB in vitro. BBB permeability was also increased, among other effects which would indicate BBB dysfunction (Hourfar et al. 2023). Similarly, in vitro and in vivo tauopathy models encountered BBB disruption and deregulation due to tau (Majerova et al. 2019).

The objective of this study was to assess whether the levels of these chosen biomarkers in the CSF or serum differ among patients with LBD (including PD and DLB), MSA, and 4RT (including CBS and PSP) compared to the control group (CG).

## Patients and methods

The study protocol was approved by the institutional ethics committee (IEC) of the Faculty of Medicine and Dentistry, Palacký University in Olomouc and University Hospital Olomouc, granted according to the University Hospital Olomouc standard SML031, and ethics committee reference numbers: 139/10 and 76/15. All patients were informed about the purpose and the design of the study and they all signed informed consent forms. All research was performed in accordance with relevant guidelines and regulations.

### Patients

The patients were consecutively recruited from 2016 to 2021 from the tertiary movement disorders outpatient clinic. Patients with the following diagnoses were included in the study: diagnosis of PD, DLB, MSA, PSP, or CBS made on the basis of validated clinical diagnostic criteria (Gilman et al. 2008; Armstrong et al. 2013; Alexander et al. 2014; Postuma et al. 2015; Boxer et al. 2017; Hoglinger et al. 2017; Bhidayasiri et al. 2019; McKeith et al., 2020). Inclusion criteria further included the ability to undergo the examination in the study protocol and adherence to follow-up. Older age brings complications and comorbidities, while sex differences also possess different risk factors. In the present study, patients with any serious disease that would interfere with the planned examination (i.e. cancer, inflammatory and haematological disease, depression, psychosis, chronic kidney disease, metabolic derangements or vascular impairment) were excluded from the study and analysis.

The control group consisted of individuals who had undergone a routine examination for benign conditions (e.g., back pain, carpal tunnel syndrome, or tension headaches), with no evidence of any neurodegenerative disease. All patients diagnosed with any of the above-mentioned neurodegenerative diseases were admitted to the ward and underwent complete clinical neurological examination according to a uniform protocol (Konicková et al. 2023). Brain magnetic resonance imaging (MRI) at 1.5T was performed in all patients and the findings were interpreted by an experienced neuroradiologist. Dopamine transporter DaTScan (123I-ioflupane) imaging was also conducted in all patients, as well as a neurophysiological examination that involved scalp electroencephalography, multimodal evoked potentials and electromyography (conduction studies and needle EMG). The patients were examined by an ophthalmologist to assess ocular abnormalities; the presence of orthostatic hypotension was tested in a dedicated laboratory. Patients were also assessed using a routine neuropsychological battery (Chertkow et al. 2019) to confirm the presence or absence of cognitive deterioration; language examination was conducted to confirm the presence or absence of aphasia or similar disorder of symbolic functions. The vascular origin of neurological symptoms, including cognitive deterioration, was excluded using imaging studies: T2-weighted, fluid-attenuated inversion recovery (FLAIR) brain MRI, diffusion-weighted MRI (DWI-MRI), ultrasonography (USG) and transcranial Doppler (TCD) examinations, and using the calculation of Hachinski ischemic score (HIS); its value in all patients was less than 3. In case of any reported urological problems, the patients were fully examined by a urologist. The diagnoses were initially formulated according to the abovementioned diagnostic criteria; all patients were followed up in the tertiary movement disorders outpatient clinic, where the final clinical diagnoses were verified using similar validated clinical diagnostic criteria at the same time as the CSF and serum examinations were conducted. From the whole cohort, four patients were autopsied, and the clinical diagnosis was confirmed in three of them.

The study participants were subsequently divided into four groups: the control group (*n* = 83) and three patient groups according to the presumed type of neurodegenerative proteinopathy: (a) patients suffering from intraneuronal synucleinopathy, i.e., LBD, which consisted of patients diagnosed with PD and DLB (*n* = 83); (b) patients suffering from glial and extra-neuronal synucleinopathy, i.e., MSA (*n* = 24); (c) patients suffering from 4RT, i.e., those diagnosed with any phenotype of PSP and CBS (*n* = 31). Sample sizes were thus selected according to the number of patients in each study group (CG, LBD, MSA and 4RT) who met the inclusion criteria, were available for CSF and/or serum analysis, and who had consented to the study. The demographic characteristics of each participant group are shown in the Results section.

### Laboratory testing

CSF samples and blood serum were collected, pre-treated, transported and stored under standardized conditions. Blood and CSF collection were performed under standard sterile conditions at 10:00 a.m. with a prior 18 h fasting period.

CSF samples were obtained from lumbar puncture, where 15 ml of CSF was collected using a 20G atraumatic spinal needle into sterile polypropylene tubes without additives. Blood serum was obtained from venipuncture (blood samples), where approximately 10 ml of peripheral blood was collected into sterile vacutainer tubes (Vranová et al. 2014, 2016; Kaleta et al. 2024). All samples were processed within 10 min of collection. Blood and CSF were centrifuged at 1100 g for 10 min at 4 °C. The serum was transferred into dark amber glass vials, heated in a water bath (30 °C for 5 min), sonicated (5 min), and bubbled with a stream of argon (2 min). CSF and serum samples were then immediately stored in the dark at − 80 °C until preparation for analysis. There was only one freeze − thaw cycle before the analysis. The biochemical analysis was conducted at the laboratory of the accredited Department of Clinical Biochemistry of the University Hospital Olomouc (CSN ISO 15189:2013; subject No. 8254; certificate No. 220/2021 valid until 9 April 2026). Biomarker levels in CSF and blood serum samples were quantified using a nephelometer (Atellica NEPH 630, Siemens Healthineers), or an Optilite turbidimeter (The Binding Site). C3 complement (C3/C3c), C4 complement (C4/C4c), haptoglobin, transferrin and orosomucoid were analysed with the nephelometer (kit manufacturer Siemens Healthineers), and β2M was analysed with the turbidimeter (kit manufacturer The Binding Site). Protein concentrations were determined based on light scattering (nephelometer) or absorbance (turbidimeter) after the formation of precipitates by the addition of analytical reagents. The CSF/serum quotients (ratios) of all biomarkers were also determined from the concentrations obtained.

### Statistical examination

The statistical software IBM SPSS Statistics version 29 (Armonk, NY: IBM Corp.) was used for data analysis and generation of figures. Logarithmic (log) transformation was used to reduce the skewness of the biomarker data for all examined biomarkers in the CSF, serum and CSF/serum quotients. Data normality was assessed in each group for each biomarker using Shapiro-Wilk tests. Results determined to be non-normal were assessed using Normal Q-Q Plots, histograms, and box plots, and had a sufficiently large enough sample size to fall under the Central Limit Theorem. As inflammation and diseases can be affected by sex and age, changes in the levels of inflammatory biomarkers for all studied groups were evaluated by Analysis of Covariance (ANCOVA) with Bonferroni-adjusted post-hoc testing, conducted using the group as the independent variable (CG, LBD, MSA and 4RT), the CSF biomarkers, serum biomarkers, or their quotients as the dependent variables (assessed individually) (C3 complement, C4 complement, haptoglobin, transferrin, orosomucoid and β2M), and age and sex as covariates. This allowed for the effect of the covariates on biomarker levels to be controlled for in the Bonferroni-adjusted post-hoc pairwise comparisons that followed. Age profiles of patient groups were compared against the CG using ANOVA with Dunnett’s post-hoc tests, while a Chi-squared test with Bonferroni’s correction of significance was used to compare the patient groups with the CG in sex distribution. Receiver operating characteristic (ROC) analysis was based on both the original log data and predicted probabilities generated by combining variables (biomarker data, sex and age) using binary logistic regression to adjust for covariates. Binary logistic regression was also used to create predicted probabilities from combinations of biomarkers between LBD vs. CG, MSA vs. CG, and 4RT vs. CG, and the same combinations of biomarkers with covariates (sex, age), which were then used to produce ROC curves. Combinations included all CSF and serum biomarkers, all CSF biomarkers, all serum biomarkers, and all significant biomarkers from the ANCOVA. The output of the logistic regressions indicated which biomarkers in these combinations may have a larger impact on the model, and thus better predictors when determining which group a patient belongs to (disease vs. control). All tests were performed at the 0.05 level of significance, and were two-sided to determine a difference in either direction.

## Results

A total of 228 individuals were included in the study; LBD patients (*n* = 83; CSF = 83, serum = 59), MSA patients (*n* = 24; CSF = 24, serum = 18), 4RT patients (*n* = 31; CSF = 31, serum = 16) and CG (*n* = 83; CSF = 83, serum = 75). Four patients were autopsied, and the clinical diagnosis was confirmed in three of them. The demographics of the cohorts are presented in Tables 1 and 2 as mean and standard deviation (SD) with minimum and maximum values for age, and as number and percentage for sex, for every study group, with the p value for the comparison of each patient group to the CG. Table 1 also shows the mean and SD with minimum and maximum values for disease duration. Log transformation was used to reduce the skewness of the biomarker data. Changes in the levels of inflammatory biomarkers for all studied groups were evaluated by ANCOVA followed by pairwise comparisons with Bonferroni-adjusted post-hoc testing.

**Table 1:** Age and disease duration demographics of study groups, including P values from age comparisons between patient groups and CG

Age (Years)	LBD	MSA	4RT	CG
Mean	66.7	67.3	67.5	57.5
SD	11.2	7.4	8.0	11.4
P value (compared to CG)	**< 0.001**	**< 0.001**	**< 0.001**	-
Minimum	38.0	52.0	51.0	24.0
Maximum	89.0	80.0	83.0	88.0

Disease Duration (Years)	LBD	MSA	4RT	-
Mean	4.27	3.83	2.55	-
SD	3.28	1.93	1.65	-
Minimum	0	1	1	-
Maximum	15	7	7	-

The mean, standard deviation (SD), minimum and maximum ages of patients in each experimental group, with p values from the comparisons between the mean of each patient group and the CG using ANOVA with Dunnett’s post-hoc tests. The mean, SD, minimum and maximum for disease duration of each patient group at the time of sampling. Results in bold are considered significant, where *p* < 0.05. LBD = Lewy Body Disease, MSA = Multiple System Atrophy, 4RT = 4-Repeat Tauopathy, CG = Control Group

**Table 2:** Sex demographics of study groups including P values from comparisons between patient groups and CG

Sex	LBD		MSA		4RT		CG		LBD vs. CG	MSA vs. CG	4RT vs. CG
	Number	Percent	Number	Percent	Number	Percent	Number	Percent			
Female	47	56.6%	19	79.2%	17	54.8%	32	38.6%	0.06	**0.001**	0.354
Male	36	43.4%	5	20.8%	14	45.2%	51	61.4%			

Number and percentage of (self-reported) females and males in each experimental group, with p values from the comparisons between each patient group and the CG using Chi-squared tests with Bonferroni’s correction. Results in bold are considered significant, where *p* < 0.05. LBD = Lewy Body Disease, MSA = Multiple System Atrophy, 4RT = 4-Repeat Tauopathy, CG = Control Group

The ANCOVA revealed that both age and sex covariates are significant in determining the dependent variable (biomarker level) for all significant CSF biomarkers, as well as for the significant CSF/serum quotients (please see Online Resource 1, Supplementary Tables 1–4 for detailed tables of the ANCOVA analyses for these biomarkers). Of the significant serum biomarkers, age and sex do not significantly affect the level of serum orosomucoid. Sex, but not age, significantly affects the level of serum C3 complement, while age, but not sex, significantly affects the level of serum β2M (please see Online Resource 1, Supplementary Tables 2, 3 and 5 for detailed tables of the ANCOVA analyses for these biomarkers).

Biomarker levels and their CSF/serum quotients were compared between each patient group and the CG, and between patient groups to each other. The pairwise comparison results are presented in Table 3, with significant results also displayed as ROC curves (please see Table 4 for the ROC analysis parameters).

### Lewy body disease (LBD)

**Fig. 1:**
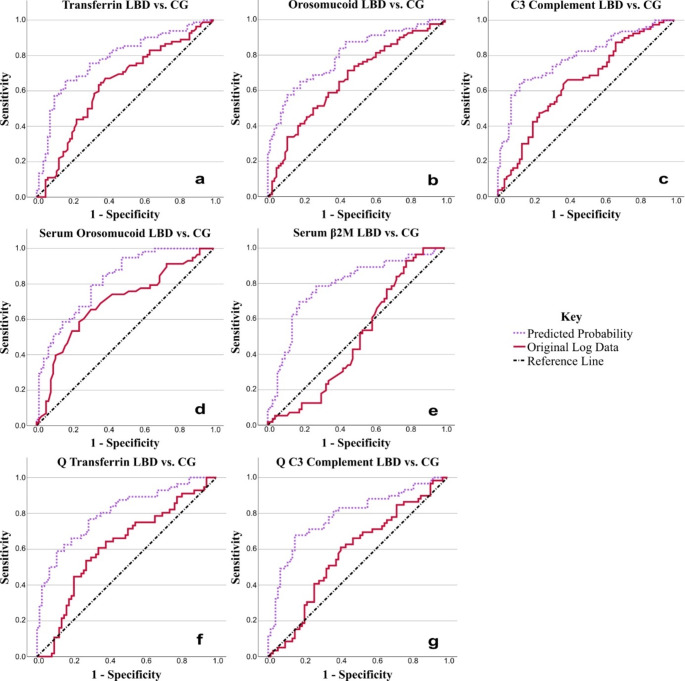
ROC curves for statistically important results from the comparison of LBD vs. CG using ANCOVA. Results from blood serum and quotients are indicated by the word serum (**d**, **e**) and Q (**f**, **g**), respectively. CSF results are the names of the biomarkers only (**a**, **b**, **c**). See Table 4 for areas under the curve. The solid red line is the ROC curve created using the original (log) data, and the dotted purple line is the ROC curve created using the predicted probabilities produced from the combination of variables (biomarker data, sex and age) in binary logistic regression. The straight diagonal dash and dot black line is the reference line. In parts **a**, **b**, **c**, **d**, **f** and **g**, the original log data curve is to the left of the reference line, with the predicted probability curve also to the left and giving a better model. In part **e**, the original log data curve crosses the reference line, but the predicted probability curve remains to the left, giving a better model. Positive actual state = LBD. LBD = Lewy Body Disease, CG = Control Group, β2M = β2 Microglobulin, Q = Quotient

In the group of LBD patients, compared to CG, there were significantly lower values of transferrin, orosomucoid and C3 complement in the CSF, and significantly lower values of orosomucoid and β2M in the serum. The CSF/serum quotient of transferrin and C3 complement were significantly lower in the LBD group than in the CG. Please see Table 3; Fig. 1.

**Table 3:** P values from the pairwise comparisons of each biomarker between all study groups

Biomarker	Type	LBD vs. CG	MSA vs. CG	4RT vs. CG	LBD vs. 4RT	MSA vs. 4RT	LBD vs. MSA
Transferrin	CSF	**0.008**	**0.007**	1	1	0.339	1
	Serum	1	1	0.821	0.903	0.315	1
	Quotient	**< 0.001**	**0.002**	0.304	1	0.949	1
Orosomucoid	CSF	**< 0.001**	**0.020**	1	*0.079*	0.262	1
	Serum	**< 0.001**	0.528	1	*0.051*	0.970	1
	Quotient	0.152	1	1	1	1	1
Haptoglobin	CSF	*0.053*	0.433	1	1	1	1
	Serum	1	1	1	1	1	1
	Quotient	1	1	0.319	1	1	1
C3 Complement	CSF	**< 0.001**	**0.002**	0.230	1	0.710	1
	Serum	0.188	1	0.140	**0.001**	**0.030**	1
	Quotient	**0.039**	*0.070*	0.284	1	1	1
C4 Complement	CSF	1	**0.027**	1	1	0.681	0.150
	Serum	1	1	1	1	1	1
	Quotient	1	0.835	1	1	1	0.600
β2M	CSF	0.248	1	1	1	1	1
	Serum	**0.028**	0.878	0.128	1	1	1
	Quotient	0.462	1	0.426	1	1	1

Comparison of biomarker concentrations between all study groups using ANCOVA, followed by pairwise comparisons with Bonferroni post-hoc testing. Each biomarker type was analysed independently (i.e. CSF Transferrin, or Serum Transferrin, or Transferrin Quotient) and then compared for each group combination (i.e. each row is one analysis). β2M = β2 microglobulin, CG = Control Group, CSF = Cerebrospinal Fluid, LBD = Lewy Body Disease, MSA = Multiple System Atrophy, 4RT = 4-Repeat Tauopathy. P values are shown in bold for statistically significant results (*p* < 0.05). P values are in italics for results trending towards statistical significance (*p* < 0.1)

Several logistic regressions were also conducted to assess the effect of combinations of biomarkers (all biomarkers, CSF biomarkers, serum biomarkers and significant biomarkers from the ANCOVA), with and without covariates (sex and age), on the likelihood of having LBD compared to CG. The overall model was statistically significant when compared to the null model (*p* < 0.05) in all combinations for LBD vs. CG, with between 19.6 and 55.8% variation explained by the model, and between 64.2 and 79.2% of cases correctly predicted. Every model was improved by the inclusion of age and sex covariates.

The best model for variation and predicted cases without covariates was all biomarkers (chi-squared (12) = 38.422, *p* < 0.001), which explained 38.5% of the variation, and correctly predicted 72.6% of cases. C3 Complement (*p* = 0.024), C4 complement (*p* = 0.029) and serum orosomucoid (*p* = 0.034) were significant and had the largest impact on the model, with serum haptoglobin (*p* = 0.090) trending towards significant. With covariates (chi-squared (14) = 61.072, *p* < 0.001), this increased to 55.8% of the variation and 76.1% correctly predicted cases. C3 complement (*p* = 0.010) and age (*p* < 0.001) were significant and had the largest impact on the model, with serum orosomucoid (*p* = 0.056) trending towards significant.

Please see Online Resource 2, Supplementary Tables 1–8, 23 for statistical details and for which biomarkers had the largest impact on the model within each combination. Please see Online Resource 2, Supplementary Fig. 1 and Supplementary Table 24 for ROC curves produced from the predicted probabilities from the logistic regressions and their parameters.

**Table 4:** ROC curve analysis parameters

Test Result Variables		Area Under the Curve	Standard Error	Asymptotic P Value	Asymptotic 95% Confidence Interval	
					Lower Bound	Upper Bound
LBD vs. CG	CSF Transferrin	0.634	0.044	**0.002**	0.547	0.720
	CSF Transferrin PP	0.780	0.037	**< 0.001**	0.708	0.852
	CSF Orosomucoid	0.659	0.043	**< 0.001**	0.575	0.742
	CSF Orosomucoid PP	0.796	0.035	**< 0.001**	0.728	0.864
	CSF C3 Complement	0.646	0.043	**0.001**	0.561	0.731
	CSF C3 Complement PP	0.791	0.036	**< 0.001**	0.721	0.861
	Serum Orosomucoid	0.694	0.047	**< 0.001**	0.602	0.786
	Serum Orosomucoid PP	0.819	0.035	**< 0.001**	0.749	0.888
	Serum β2M	0.487	0.051	0.799	0.387	0.587
	Serum β2M PP	0.786	0.042	**< 0.001**	0.704	0.868
	Q Transferrin	0.616	0.050	**0.021**	0.517	0.715
	Q Transferrin PP	0.804	0.039	**< 0.001**	0.727	0.880
	Q C3 Complement	0.578	0.050	0.120	0.480	0.676
	Q C3 Complement PP	0.786	0.041	**< 0.001**	0.705	0.866
MSA vs. CG	CSF Transferrin	0.703	0.061	**0.001**	0.584	0.822
	CSF Transferrin PP	0.881	0.039	**< 0.001**	0.804	0.957
	CSF Orosomucoid	0.671	0.059	**0.004**	0.556	0.787
	CSF Orosomucoid PP	0.866	0.045	**< 0.001**	0.777	0.955
	CSF C3 Complement	0.716	0.058	**< 0.001**	0.602	0.830
	CSF C3 Complement PP	0.888	0.039	**< 0.001**	0.812	0.964
	CSF C4 Complement	0.658	0.061	**0.010**	0.538	0.777
	CSF C4 Complement PP	0.866	0.043	**< 0.001**	0.781	0.951
	Q Transferrin	0.724	0.059	**< 0.001**	0.609	0.839
	Q Transferrin PP	0.906	0.032	**< 0.001**	0.843	0.969
LBD vs. 4RT	Serum C3 Complement	0.796	0.067	**< 0.001**	0.666	0.926
	Serum C3 Complement PP	0.798	0.067	**< 0.001**	0.666	0.929
MSA vs. 4RT	Serum C3 Complement	0.754	0.086	**0.003**	0.585	0.922
	Serum C3 Complement PP	0.759	0.084	**0.002**	0.595	0.924

**Fig. 2:**
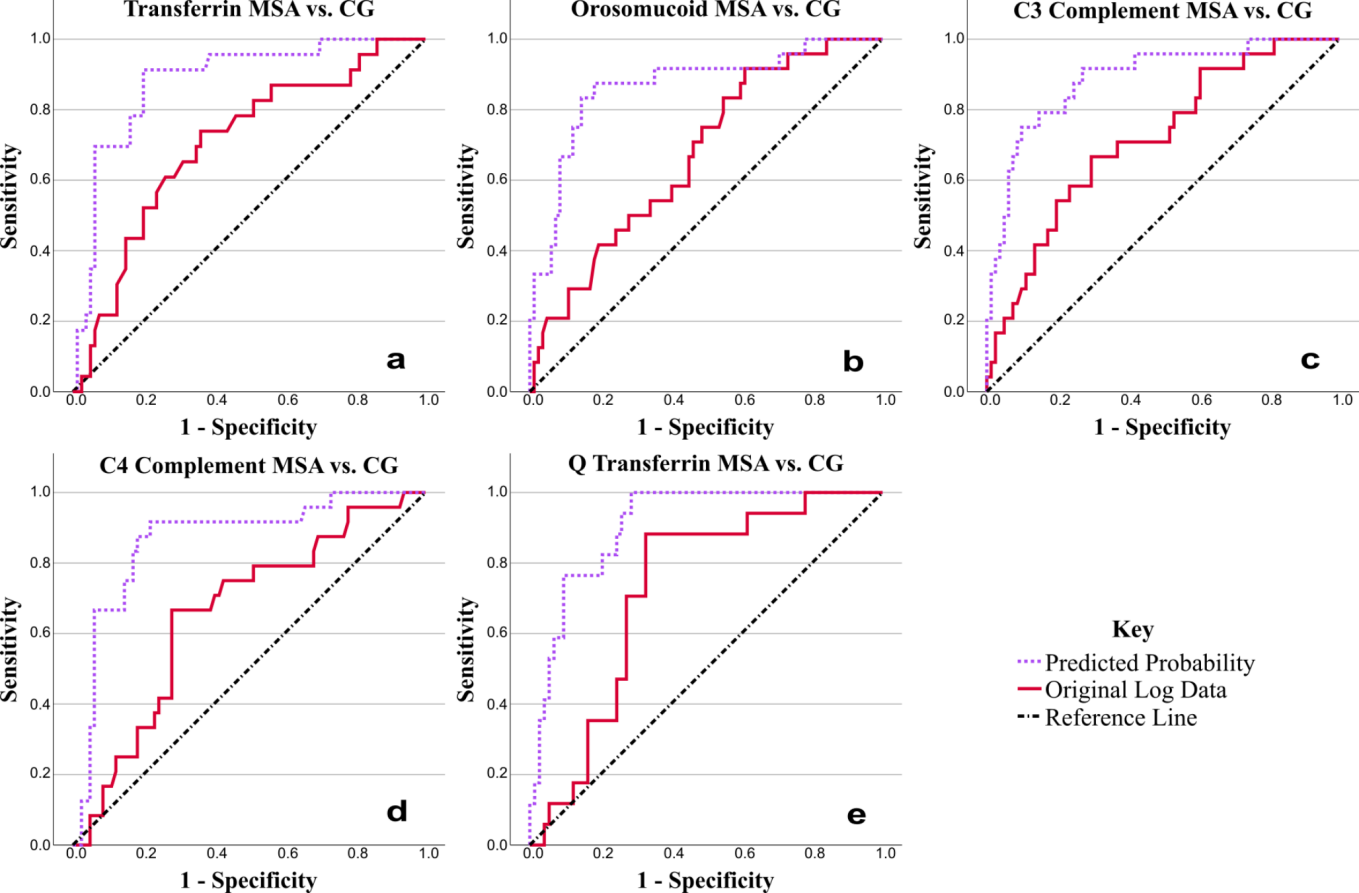
ROC curves for statistically important results from the comparison of MSA vs. CG using ANCOVA. Results from quotients are indicated by the letter Q (**e**), CSF results are the names of the biomarkers only (**a**, **b**, **c**, **d**). See Table 4 for areas under the curve. The solid red line is the ROC curve created using the original (log) data, and the dotted purple line is the ROC curve created using the predicted probabilities produced from the combination of variables (biomarker data, sex and age) in binary logistic regression. The straight diagonal dash and dot black line is the reference line. In all parts, the original log data curve is to the left of the reference line, with the predicted probability curve also to the left and giving a better model. Positive actual state = MSA. MSA = Multiple System Atrophy, CG = Control Group, Q = Quotient

**Fig. 3:**
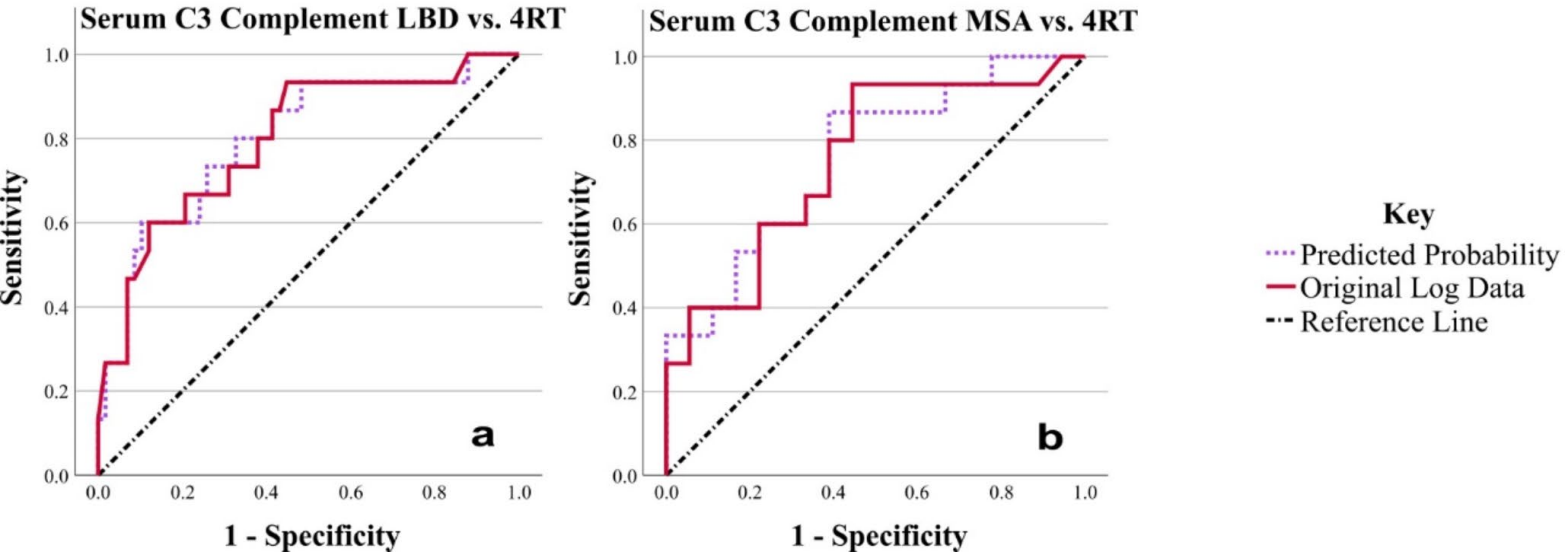
ROC curves for statistically important results from comparison of LBD and MSA vs. 4RT using ANCOVA. Results from blood serum are indicated by the word serum (**a**, **b**). See Table 4 for areas under the curve. The solid red line is the ROC curve created using the original (log) data, and the dotted purple line is the ROC curve created using the predicted probabilities produced from the combination of variables (biomarker data, sex and age) in binary logistic regression. The straight diagonal dash and dot black line is the reference line. In both parts, the original log data and predicted probability curves are to the left of the reference line, almost overlapping. Positive actual state = 4RT. LBD = Lewy Body Disease, MSA = Multiple System Atrophy, 4RT = 4-Repeat Tauopathy

The area under the curve, standard error, asymptotic P value, and asymptotic 95% Confidence Intervals of each ROC curve from Figs. 1, 2 and 3 (original log data and predicted probabilities (PP)). P values are shown in bold for statistically significant results (*p* < 0.05). β2M = β2 microglobulin, CG = Control Group, CSF = Cerebrospinal Fluid, LBD = Lewy Body Disease, MSA = Multiple System Atrophy, Q = Quotient, 4RT = 4-Repeat Tauopathy

### Multiple system atrophy (MSA)

Significantly lower values of transferrin, C3 complement, C4 complement and orosomucoid were demonstrated in MSA compared to the CG in the CSF. The CSF/serum quotient of transferrin was significantly lower in the MSA group than in the CG. Please see Table 3; Fig. 2.

Several logistic regressions were also conducted to assess the effect of combinations of biomarkers (all biomarkers, CSF biomarkers, serum biomarkers and significant biomarkers from the ANCOVA), with and without covariates (sex and age), on the likelihood of having MSA compared to CG. The overall model was statistically significant when compared to the null model (*p* < 0.05) in six out of eight combinations for MSA vs. CG, with between 25.5 and 57.8% variation explained by the significant models, and between 80.5 and 87.5% of cases correctly predicted in the significant models. Every model was improved by the inclusion of age and sex covariates.

The best model for variation and predicted cases without covariates was all biomarkers (chi-squared (12) = 23.055, *p* = 0.027), which explained 39.4% of the variation, and correctly predicted 84.6% of cases. C3 Complement (*p* = 0.019), orosomucoid (*p* = 0.010), β2M (*p* = 0.010), serum haptoglobin (*p* = 0.024) and serum orosomucoid (*p* = 0.007) were significant and had the largest impact on the model, with transferrin (*p* = 0.096) trending towards significant. With covariates (chi-squared (14) = 33.769, *p* = 0.002), this increased to 54.1% of the variation and 85.9% correctly predicted cases. C3 Complement (*p* = 0.047), serum orosomucoid (*p* = 0.037) and age (*p* = 0.017) were significant and had the largest impact on the model, with orosomucoid (*p* = 0.069) and serum haptoglobin (*p* = 0.078) trending towards significant.

Please see Online Resource 2, Supplementary Tables 9–16, 23 for statistical details and for which biomarkers had the largest impact on the model within each combination. Please see Online Resource 2, Supplementary Fig. 2 and Supplementary Table 24 for ROC curves produced from the predicted probabilities from the logistic regressions and their parameters.

### 4-repeat tauopathies (4RT)

There were no significant differences between 4RT and the CG, however, significant differences were detected between the synucleinopathy patient groups (LBD and MSA) and the 4RT patient group in the serum. These included a significantly lower level of C3 complement in LBD and MSA compared to 4RT, and a strong trend towards lower levels of orosomucoid in LBD compared to 4RT. Please see Table 3; Fig. 3.

Several logistic regressions were conducted to assess the effect of combinations of biomarkers (all biomarkers, CSF biomarkers and serum biomarkers), with and without covariates (sex and age), on the likelihood of having 4RT compared to CG. The overall model was statistically significant when compared to the null model (*p* < 0.05) in four out of six combinations for 4RT vs. CG, with between 35.9 and 61.5% variation explained by the significant models, and between 75.5 and 90.5% of cases correctly predicted in the significant models. Every model was improved by the inclusion of age and sex covariates.

The best model for variation and predicted cases without covariates was all biomarkers (chi-squared (12) = 22.089, *p* = 0.037), which explained 42.6% of the variation, and correctly predicted 86.5% of cases. Haptoglobin (*p* = 0.009), serum haptoglobin (*p* = 0.031) and serum C3 complement (*p* = 0.011) were significant and had the largest impact on the model, with C3 complement (*p* = 0.050) and orosomucoid (*p* = 0.051) trending towards significant. With covariates (chi-squared (14) = 34.419, *p* = 0.002), this increased to 61.5% of the variation and 90.5% correctly predicted cases. Haptoglobin (*p* = 0.033), serum C3 complement (*p* = 0.030) and age (*p* = 0.013) were significant and had the largest impact on the model, with serum haptoglobin (*p* = 0.058) trending towards significant.

Please see Online Resource 2, Supplementary Tables 17–23 for statistical details and for which biomarkers had the largest impact on the model within each combination. Please see Online Resource 2, Supplementary Fig. 3 and Supplementary Table 24 for ROC curves produced from the predicted probabilities from the logistic regressions and their parameters.

All of the remaining CSF and serum biomarkers, and quotients were found to be non-significant.

The ROC curves (Figs. 1, 2 and 3) and their analysis parameters (Table 4) show that while the original log data of the patients creates a fair model for the discrimination between groups for many of the biomarkers, when the covariates are adjusted for by using binary logistic regression and the resulting predicted probabilities, the model produced is better in discriminating between groups for all biomarkers, particularly for β2M (Fig. 1).

Additionally, fold changes were calculated for the relative differences of the estimated marginal means from the ANCOVA analysis between the patient groups and the CG. These are presented in Table 5 and shows the percentage changes between the groups.

**Table 5:** Fold changes of biomarkers between patient groups and CG in percentage

Biomarkers	CSF			Serum			Q		
	LBD	MSA	4RT	LBD	MSA	4RT	LBD	MSA	4RT
Transferrin	**-16.05**	**-23.09**	-8.59	0.46	-3.39	6.91	**-18.72**	**-24.14**	-13.70
Orosomucoid	**-24.32**	**-25.18**	-6.89	**-16.44**	-10.87	0.23	-13.30	-9.22	-8.38
Haptoglobin	-28.71	-29.21	-20.93	-4.50	-2.95	4.71	-15.28	-9.64	-29.21
C3 Complement	**-23.44**	**-29.69**	-16.82	-7.32	-5.81	12.72	**-18.53**	-23.97	-19.65
C4 Complement	-5.81	**-20.93**	-8.80	-5.59	-9.22	4.23	0.93	-12.10	-5.81
β2M	-9.43	-5.59	-5.38	**-13.10**	-9.84	-15.08	11.94	7.65	17.76

Values are expressed as relative differences of the estimated marginal means from ANCOVA analysis (in percentages) between patient groups and the CG. Bold values are statistically significant in ANCOVA. β2M = β2 microglobulin, CG = Control Group, CSF = Cerebrospinal Fluid, LBD = Lewy Body Disease, MSA = Multiple System Atrophy, Q = Quotient, 4RT = 4-Repeat Tauopathy

## Discussion

The present study broadens the list of biomarkers that can be investigated for different diseases, while highlighting aspects related to the pathogenesis of parkinsonian diseases. While biomarkers and diagnostic criteria exist for neurodegenerative diseases, with acceptable sensitivity and specificity, there is difficulty in their diagnosis due to the considerable overlap between similar diseases. Reliable, non-invasive, fluid biomarkers would be ideal in the search for additional biomarkers for the diagnosis and prognosis of neurodegenerative proteinopathies. Considering their devastating impact, it would be highly beneficial to identify inflammatory biomarkers which are different in MSA, LBD and 4RT patients compared to healthy controls, as well as to each other.

It was observed that the CSF concentrations of all biomarkers were reduced in all three cohorts (LBD, MSA, 4RT) when comparing to CG (Table 5) but the concentration changes were only moderate, and only for some biomarkers achieved statistical significance, particularly for LBD and MSA. No significance was detected for 4RT. In contrast the serum concentrations of tested biomarkers were not uniform. There was a preferential decrease detected in the group of LBD and MSA, and a slight increase in 4RT, but statistical significance was achieved only for orosomucoid and β2M in the LBD cohort.

Several models were evaluated, whereby individual biomarkers and combinations of biomarkers were assessed, with and without covariates. The inclusion of covariates was always beneficial to the model, and while individual biomarker changes were significant, combinations of biomarkers may also be advantageous (please see Figs. 1, 2 and 3; Tables 3 and 4, Online Resource 1, and Online Resource 2). Both types of model could potentially be applied by future research and diagnostics.

Results indicate that systemic inflammatory activation is only modest, if any, and local brain-focused inflammation is of limited capacity to activate systemic response, including that contributing to immunomodulation. It does not mean that modest local inflammation could not deteriorate brain structure and function. Conversely, the analyses revealed modest, yet significant changes in selected acute response proteins in both CSF and serum, possibly offering a valuable future tool for fluid-based differential diagnostics of LBD, MSA, and 4RT.

### C3/C4 complement

In the present study, C3 complement (C3/C3c) levels were significantly decreased in LBD and MSA patients compared to the CG, and C4 complement (C4/C4c) levels were significantly decreased in MSA patients compared to the CG (see Table 3), likely reflecting activation of the complement system and C3/C4 complement consumption. This is in accordance with studies reporting an increase in C3/C4 fragments or decrease in full length C3/C4 for PD (Yamada et al. 1992; Loeffler et al. 2006; Gregersen et al. 2021) and MSA patients (Wang et al. 2011). It was also revealed that C3 complement was significantly reduced in LBD and MSA compared to 4RT in the serum. This suggests that the different pathologies present within the CNS may lead to different physiological responses, resulting in biomarker differences. A decrease in full length C3/C4 complement is likely from complement pathway activation and cleavage into fragments (Thurman and Yapa 2019). The C3 complement quotient is the ratio of CSF C3 to serum C3, and could be indicative of a potential disruption of the BBB and/or C3 complement transport (Asano et al. 2017), or as a result of complement activation in the CNS and not/less in the periphery. A significant difference was found between the LBD patient group and CG of the present study, where it was decreased, with a strong trend towards a decreased quotient between the MSA patient group and CG, which could suggest that synucleinopathy patients have a larger BBB and/or C3 complement transport disruption, or CSF complement activation than 4RT tauopathy patients (Song et al. 2011; Al-Bachari et al. 2020).

### Orosomucoid

The results of the present study show a decreased level of orosomucoid in MSA and LBD patients compared to the CG in the CSF, and in LBD compared to the CG in the serum, as well as a strong trend towards lower levels in LBD compared to 4RT in the serum (see Table 3). One possibility for a lower concentration may be its consumption or sequestration during anti-inflammatory functions (Jo et al. 2017), where a deficiency could also be detrimental. This could also correlate with the downregulated orosomucoid found in the amygdala in PD by Villar-Conde et al. (Villar-Conde et al. 2023).

The change in orosomucoid concentration does not occur in 4RT patients compared to CG, which may be related to the candidate protein involved (α-syn in MSA and LBD, and tau protein in 4RT).

### Transferrin

In the present study, Tf was found to be decreased in the CSF, but not the serum, of MSA and LBD patients compared to the CG. Some studies have found Tf concentrations in the circulation (Logroscino et al. 1997) and brain tissue (substantia nigra (Ayton et al. 2016) and temporal cortices (Sabbir 2024)) to be significantly lower in PD patients compared to the CG, while Xu et al. (2018) found increased Tf levels in the serum of PD patients (Xu et al. 2018). Both suggest abnormal Tf trafficking and/or iron metabolism in PD but results are inconsistent.

The Tf quotient is the ratio of CSF Tf to serum Tf, and could be indicative of a potential disruption of the BBB and/or Tf transport (Asano et al. 2017), or the consumption/occupation of the protein. A significant difference was found in the LBD and MSA synucleinopathy patient groups of the present study, where it was decreased, which could suggest that synucleinopathy patients have a larger BBB and/or Tf transport disruption, or higher consumption/occupation of the protein, than 4RT tauopathy patients (Zeman et al. 2000; Song et al. 2011; Ayton et al. 2016; Al-Bachari et al. 2020).

### Haptoglobin

The present study revealed a non-significant trend towards decreased CSF levels of haptoglobin in LBD compared to the CG (*p* = 0.053 (Table 3), please see Online Resource 1, Supplementary Table 6 for detailed ANCOVA analyses). A number of studies suggest that either higher haptoglobin concentrations could be protective (Costa-Mallen et al. 2016), or lower haptoglobin may be a risk factor and/or indicator of neurodegeneration (Cocciolo et al. 2012; Chaubey et al. 2016).

### Β2 microglobulin

The present study found significantly decreased levels of β2M in LBD patients compared to the CG in the serum (*p* = 0.028). Decreased β2M in the CSF was previously reported in PD (Mogi et al. 1989). There is an overall decrease in the estimated marginal mean of β2M across each patient group compared to the CG (as seen in Table 5), but only LBD vs. CG is significant. Although the lower level could possibly be due to β2M interaction with α-syn (Rani et al. 2023), it seems that both local β2M sequestration and replenishment contribute to the detected concentration, as evidenced from discrepancies between serum and CSF β2M concentrations. β2M concentration was found by others to be dynamic in AD, changing depending on disease stage (Sheng et al. 2025). The ROC curve of β2M (Fig. 1) shows that inclusion of covariates greatly improved discrimination between groups, reflecting the large influence of covariates in β2M analysis.

## Limitations, future research and conclusions

In the majority of patients, the clinical diagnoses were conducted at the highest level of probability, and were pathologically confirmed in three patients; this unavoidable limitation would only be removed when the brains of all patients are available for assessment.

It should also be taken into consideration that a significantly higher age was demonstrated in the patient groups compared to the CG, as well as a significantly higher proportion of women in the MSA group. Older individuals, and an increased proportion of women to men, is a widely known phenomenon previously observed in community DLB cohorts (Chiu et al. 2023), and so is not specific to the cohorts in the present study only. Older age may present with low levels or stages of chronic disease conditions (with variations due to sex) which may not have been identified and could interfere with the results. While this has been carefully controlled for, it still exists as a limitation of the study, and future studies will need to confirm the results in precisely age- and sex-matched subjects. Further research will be essential in the search for biomarkers for the diagnosis and prognosis of LBD, MSA, and 4RT.

Future research should analyse the CSF and serum in a new cohort of patients and compare the results with the current study; these results should then be compared with confirmed clinical diagnoses (at the time and in the future, including autopsies) to determine accuracy. Furthermore, the results of the present study raise the question of whether the inflammation is upstream, downstream, or both of cell damage. Is inflammation an initiating factor for cell damage, or is cell damage an inducer of inflammation? In chronic diseases there will inevitably be a feedback loop where both are true, but which comes first? That is a more difficult question to answer. This could be investigated in future research through the use of cellular models, including organoids, and/or animal models, with the application of cytokines, biomarkers, inflammatory milieu (conditioned media, serum, etc.) and/or aggregated proteins. Both healthy and disease models, with or without mutations, could be used. In terms of clinical probands, causal mutation carriers could be followed and monitored for biomarker changes through life. As this study only looks at a snapshot of disease at one point in time, it would be necessary for future research to include longitudinal studies, to evaluate biomarker levels and their stability throughout the course of the disease.

With ongoing research in this field, it has been increasingly suggested that the inflammatory process may have crucial consequences on neurodegeneration (Kwon and Koh 2020; Mishra et al. 2021; Balistreri and Monastero 2023; Zhang et al. 2023; Adamu et al. 2024; Giri et al. 2024). The results of the present study indicate that there are a variety of biochemical differences between parkinsonian disorders and the healthy state, and that the inflammatory process in neurodegeneration is reflected in the CSF and serum. This was shown by the significantly decreased values in CSF biomarkers (LBD: transferrin, C3 complement, orosomucoid; MSA: transferrin, C3 complement, C4 complement, orosomucoid), as well as serum biomarkers (LBD: orosomucoid, β2M) in patient groups compared to the CG. The results also revealed significant differences between the synucleinopathy patient groups and the tauopathy patient group in the serum (LBD and MSA: C3 complement), suggesting that the type of pathology present in the CNS may influence these biomarkers. Further research is required to answer such questions as: which pathways are involved in neuroinflammation and how this occurs, what molecules are involved and where they come from, and which biomarkers could potentially be useful in future research and diagnostics.

## Electronic supplementary material

Below is the link to the electronic supplementary material.

Supplementary Material 1

Supplementary Material 2

## Data Availability

Detailed information is provided in the tables (Tables 1, 2, 3, 4 and 5; Online Resource 1, Supplementary Tables 1–6; Online Resource 2, Supplementary Tables 1–24) used in this study. Datasets are available from the corresponding author upon reasonable request: The raw data supporting the conclusions of this article will be made available by the authors, without undue reservation.

## Abbreviations

4RT: 4-repeat tauopathies
α-syn: Alpha-synuclein
ANCOVA: Analysis of Covariance
BBB: Blood brain barrier
β2M: β2 microglobulin
CBS: Corticobasal syndrome
CG: Control group
CNS: Central nervous system
CSF: Cerebrospinal fluid
DLB: Dementia with Lewy bodies
DWI-MRI: Diffusion-weighted MRI
FLAIR: Fluid-attenuated inversion recovery
HIS: Hachinski ischemic score
IEC: Institutional ethics committee
LBD: Lewy body diseases
Log: Logarithmic
MRI: Magnetic resonance imaging
MSA: Multiple system atrophy
PD: Parkinson’s disease
PNS: Peripheral nervous system
PSP: Progressive supranuclear palsy
Q: Quotient
ROC: Receiver operating characteristic
SD: Standard deviation
TCD: Transcranial Doppler
Tf: Transferrin
USG: Ultrasonography
