# Supplementary material for: Comparison of inflammatory biomarker levels in neurodegenerative proteinopathies: a case-control study
Source: J Neural Transm (Vienna). 2025 Mar 3;132(6):811–26. doi: 10.1007/s00702-025-02902-6 (PMC12116722; doi:10.1007/s00702-025-02902-6)
Supplement: Supplementary file 1 — Supplementary Material 1 [file 702_2025_2902_MOESM1_ESM.docx]

Online Resource 1

Comparison of inflammatory biomarker levels in neurodegenerative proteinopathies: a case-control study

*Journal of Neural Transmission*

**Sarah E. V. Cook^1, 2 *^, Kateřina Menšíková, Dorota Koníčková, Hedvika Šlanhofová, Kateřina Klíčová, Milan Raška, Jana Zapletalová, David Friedecký, Petr Kaňovský**

^1^Department of Neurology, University Hospital Olomouc, Olomouc, Czech Republic

^2^Department of Neurology, Faculty of Medicine and Dentistry, Palacký University, Olomouc, Czech Republic

^*^ Corresponding Author: Sarah Cook ([sarah.cook01@upol.cz](mailto:sarah.cook01@upol.cz)) (ORCID: 0000-0002-4389-947X)

Supplementary Table 1. ANCOVA analyses for Transferrin

| **ANCOVA Analyses** | | | | | | | |
| --- | --- | --- | --- | --- | --- | --- | --- |
| Dependent Variable: Transferrin | | | | | | | |
| **CSF** | **Source** | **Type III Sum of Squares** | **df** | **Mean Square** | **F** | **Sig.** | **Partial Eta Squared** |
|  | **Corrected Model** | 0.534^a^ | 5 | 0.107 | 5.611 | **<0.001** | 0.119 |
|  | **Intercept** | 7.321 | 1 | 7.321 | 384.611 | **<0.001** | 0.649 |
|  | **Age** | 0.174 | 1 | 0.174 | 9.135 | **0.003** | 0.042 |
|  | **Sex** | 0.088 | 1 | 0.088 | 4.62 | **0.033** | 0.022 |
|  | **Group** | 0.292 | 3 | 0.097 | 5.11 | **0.002** | 0.069 |
|  | **Error** | 3.959 | 208 | 0.019 |  |  |  |
|  | **Total** | 393.986 | 214 |  |  |  |  |
|  | **Corrected Total** | 4.493 | 213 |  |  |  |  |
|  | a. R Squared = 0.119 (Adjusted R Squared = 0.098) | | | | | | |
| **Serum** | **Corrected Model** | 0.154^b^ | 5 | 0.031 | 7.204 | **<0.001** | 0.183 |
|  | **Intercept** | 1.142 | 1 | 1.142 | 266.545 | **<0.001** | 0.623 |
|  | **Age** | 0.106 | 1 | 0.106 | 24.793 | **<0.001** | 0.133 |
|  | **Sex** | 0.022 | 1 | 0.022 | 5.059 | **0.026** | 0.03 |
|  | **Group** | 0.017 | 3 | 0.006 | 1.302 | 0.276 | 0.024 |
|  | **Error** | 0.69 | 161 | 0.004 |  |  |  |
|  | **Total** | 25.71 | 167 |  |  |  |  |
|  | **Corrected Total** | 0.844 | 166 |  |  |  |  |
|  | b. R Squared = 0.183 (Adjusted R Squared = 0.157) | | | | | | |
| **Quotient** | **Corrected Model** | 0.846^c^ | 5 | 0.169 | 12.643 | **<0.001** | 0.287 |
|  | **Intercept** | 1.836 | 1 | 1.836 | 137.164 | **<0.001** | 0.466 |
|  | **Age** | 0.472 | 1 | 0.472 | 35.271 | **<0.001** | 0.183 |
|  | **Sex** | 0.186 | 1 | 0.186 | 13.873 | **<0.001** | 0.081 |
|  | **Group** | 0.288 | 3 | 0.096 | 7.179 | **<0.001** | 0.121 |
|  | **Error** | 2.102 | 157 | 0.013 |  |  |  |
|  | **Total** | 153.145 | 163 |  |  |  |  |
|  | **Corrected Total** | 2.948 | 162 |  |  |  |  |
|  | c. R Squared = 0.287 (Adjusted R Squared = 0.264) | | | | | | |

Table of ANCOVA analyses (age and sex covariates) with Bonferroni-adjusted post-hoc testing for Transferrin for the CSF, serum, and quotient. P values are bold for statistically significant results (p < 0.05). CSF = Cerebrospinal Fluid.

Supplementary Table 2. ANCOVA analyses for Orosomucoid

| **ANCOVA Analyses** | | | | | | | |
| --- | --- | --- | --- | --- | --- | --- | --- |
| Dependent Variable: Orosomucoid | | | | | | | |
| **CSF** | **Source** | **Type III Sum of Squares** | **df** | **Mean Square** | **F** | **Sig.** | **Partial Eta Squared** |
|  | **Corrected Model** | 1.351^a^ | 5 | 0.27 | 9.289 | **<0.001** | 0.18 |
|  | **Intercept** | 1.969 | 1 | 1.969 | 67.693 | **<0.001** | 0.243 |
|  | **Age** | 0.375 | 1 | 0.375 | 12.907 | **<0.001** | 0.058 |
|  | **Sex** | 0.352 | 1 | 0.352 | 12.091 | **<0.001** | 0.054 |
|  | **Group** | 0.613 | 3 | 0.204 | 7.021 | **<0.001** | 0.091 |
|  | **Error** | 6.137 | 211 | 0.029 |  |  |  |
|  | **Total** | 155.908 | 217 |  |  |  |  |
|  | **Corrected Total** | 7.487 | 216 |  |  |  |  |
|  | a. R Squared = 0.180 (Adjusted R Squared = 0.161) | | | | | | |
| **Serum** | **Corrected Model** | 0.198^b^ | 5 | 0.04 | 3.576 | **0.004** | 0.1 |
|  | **Intercept** | 0.08 | 1 | 0.08 | 7.174 | **0.008** | 0.043 |
|  | **Age** | 0.029 | 1 | 0.029 | 2.613 | 0.108 | 0.016 |
|  | **Sex** | 0.00002008 | 1 | 0.00002008 | 0.002 | 0.966 | 0 |
|  | **Group** | 0.194 | 3 | 0.065 | 5.829 | **<0.001** | 0.098 |
|  | **Error** | 1.787 | 161 | 0.011 |  |  |  |
|  | **Total** | 2.611 | 167 |  |  |  |  |
|  | **Corrected Total** | 1.985 | 166 |  |  |  |  |
|  | b. R Squared = 0.100 (Adjusted R Squared = 0.072) | | | | | | |
| **Quotient** | **Corrected Model** | 0.699^c^ | 5 | 0.14 | 6.872 | **<0.001** | 0.175 |
|  | **Intercept** | 2.242 | 1 | 2.242 | 110.269 | **<0.001** | 0.405 |
|  | **Age** | 0.22 | 1 | 0.22 | 10.827 | **0.001** | 0.063 |
|  | **Sex** | 0.387 | 1 | 0.387 | 19.01 | **<0.001** | 0.105 |
|  | **Group** | 0.105 | 3 | 0.035 | 1.722 | 0.165 | 0.031 |
|  | **Error** | 3.294 | 162 | 0.02 |  |  |  |
|  | **Total** | 138.492 | 168 |  |  |  |  |
|  | **Corrected Total** | 3.992 | 167 |  |  |  |  |
|  | c. R Squared = 0.175 (Adjusted R Squared = 0.150) | | | | | | |

Table of ANCOVA analyses (age and sex covariates) with Bonferroni-adjusted post-hoc testing for Orosomucoid for the CSF, serum, and quotient. P values are bold for statistically significant results (p < 0.05). CSF = Cerebrospinal Fluid.

Supplementary Table 3. ANCOVA analyses for C3 Complement

| **ANCOVA Analyses** | | | | | | | |
| --- | --- | --- | --- | --- | --- | --- | --- |
| Dependent Variable: C3 Complement | | | | | | | |
| **CSF** | **Source** | **Type III Sum of Squares** | **df** | **Mean Square** | **F** | **Sig.** | **Partial Eta Squared** |
|  | **Corrected Model** | 1.377^a^ | 5 | 0.275 | 9.572 | **<0.001** | 0.186 |
|  | **Intercept** | 1.232 | 1 | 1.232 | 42.839 | **<0.001** | 0.17 |
|  | **Age** | 0.337 | 1 | 0.337 | 11.706 | **<0.001** | 0.053 |
|  | **Sex** | 0.396 | 1 | 0.396 | 13.774 | **<0.001** | 0.062 |
|  | **Group** | 0.59 | 3 | 0.197 | 6.838 | **<0.001** | 0.089 |
|  | **Error** | 6.013 | 209 | 0.029 |  |  |  |
|  | **Total** | 112.535 | 215 |  |  |  |  |
|  | **Corrected Total** | 7.389 | 214 |  |  |  |  |
|  | a. R Squared = 0.186 (Adjusted R Squared = 0.167) | | | | | | |
| **Serum** | **Corrected Model** | 0.140^b^ | 5 | 0.028 | 4.631 | **<0.001** | 0.126 |
|  | **Intercept** | 0.152 | 1 | 0.152 | 25.168 | **<0.001** | 0.136 |
|  | **Age** | 0 | 1 | 0 | 0.05 | 0.823 | 0 |
|  | **Sex** | 0.029 | 1 | 0.029 | 4.768 | **0.030** | 0.029 |
|  | **Group** | 0.096 | 3 | 0.032 | 5.301 | **0.002** | 0.09 |
|  | **Error** | 0.968 | 160 | 0.006 |  |  |  |
|  | **Total** | 5.376 | 166 |  |  |  |  |
|  | **Corrected Total** | 1.108 | 165 |  |  |  |  |
|  | b. R Squared = 0.126 (Adjusted R Squared = 0.099) | | | | | | |
| **Quotient** | **Corrected Model** | 0.842^c^ | 5 | 0.168 | 5.861 | **<0.001** | 0.153 |
|  | **Intercept** | 0.256 | 1 | 0.256 | 8.912 | **0.003** | 0.052 |
|  | **Age** | 0.484 | 1 | 0.484 | 16.838 | **<0.001** | 0.094 |
|  | **Sex** | 0.174 | 1 | 0.174 | 6.044 | **0.015** | 0.036 |
|  | **Group** | 0.316 | 3 | 0.105 | 3.663 | **0.014** | 0.064 |
|  | **Error** | 4.656 | 162 | 0.029 |  |  |  |
|  | **Total** | 56.216 | 168 |  |  |  |  |
|  | **Corrected Total** | 5.499 | 167 |  |  |  |  |
|  | c. R Squared = 0.153 (Adjusted R Squared = 0.127) | | | | | | |

Table of ANCOVA analyses (age and sex covariates) with Bonferroni-adjusted post-hoc testing for C3 complement for the CSF, serum, and quotient. P values are bold for statistically significant results (p < 0.05). CSF = Cerebrospinal Fluid.

Supplementary Table 4. ANCOVA analyses for C4 Complement

| **ANCOVA Analyses** | | | | | | | |
| --- | --- | --- | --- | --- | --- | --- | --- |
| Dependent Variable: C4 Complement | | | | | | | |
| **CSF** | **Source** | **Type III Sum of Squares** | **df** | **Mean Square** | **F** | **Sig.** | **Partial Eta Squared** |
|  | **Corrected Model** | 0.943^a^ | 5 | 0.189 | 9.209 | **<0.001** | 0.178 |
|  | **Intercept** | 0.013 | 1 | 0.013 | 0.616 | 0.433 | 0.003 |
|  | **Age** | 0.503 | 1 | 0.503 | 24.591 | **<0.001** | 0.104 |
|  | **Sex** | 0.24 | 1 | 0.24 | 11.731 | **<0.001** | 0.052 |
|  | **Group** | 0.172 | 3 | 0.057 | 2.806 | **0.041** | 0.038 |
|  | **Error** | 4.34 | 212 | 0.02 |  |  |  |
|  | **Total** | 28.625 | 218 |  |  |  |  |
|  | **Corrected Total** | 5.283 | 217 |  |  |  |  |
|  | a. R Squared = 0.178 (Adjusted R Squared = 0.159) | | | | |  |  |
| **Serum** | **Corrected Model** | 0.151^b^ | 5 | 0.03 | 1.744 | 0.127 | 0.051 |
|  | **Intercept** | 1.012 | 1 | 1.012 | 58.583 | **<0.001** | 0.267 |
|  | **Age** | 0.003 | 1 | 0.003 | 0.193 | 0.661 | 0.001 |
|  | **Sex** | 0.064 | 1 | 0.064 | 3.705 | 0.056 | 0.022 |
|  | **Group** | 0.047 | 3 | 0.016 | 0.915 | 0.435 | 0.017 |
|  | **Error** | 2.783 | 161 | 0.017 |  |  |  |
|  | **Total** | 54.078 | 167 |  |  |  |  |
|  | **Corrected Total** | 2.933 | 166 |  |  |  |  |
|  | b. R Squared = 0.051 (Adjusted R Squared = 0.022) | | | | | | |
| **Quotient** | **Corrected Model** | 0.538^c^ | 5 | 0.108 | 5.925 | **<0.001** | 0.158 |
|  | **Intercept** | 1.269 | 1 | 1.269 | 69.87 | **<0.001** | 0.307 |
|  | **Age** | 0.376 | 1 | 0.376 | 20.675 | **<0.001** | 0.116 |
|  | **Sex** | 0.046 | 1 | 0.046 | 2.507 | 0.115 | 0.016 |
|  | **Group** | 0.059 | 3 | 0.02 | 1.074 | 0.362 | 0.02 |
|  | **Error** | 2.87 | 158 | 0.018 |  |  |  |
|  | **Total** | 129.944 | 164 |  |  |  |  |
|  | **Corrected Total** | 3.408 | 163 |  |  |  |  |
|  | c. R Squared = 0.158 (Adjusted R Squared = 0.131) | | | | | | |

Table of ANCOVA analyses (age and sex covariates) with Bonferroni-adjusted post-hoc testing for C4 complement for the CSF, serum, and quotient. P values are bold for statistically significant results (p < 0.05). CSF = Cerebrospinal Fluid.

Supplementary Table 5. ANCOVA analyses for β2M

| **ANCOVA Analyses** | | | | | | | |
| --- | --- | --- | --- | --- | --- | --- | --- |
| Dependent Variable: β2M | | | | | | | |
| **CSF** | **Source** | **Type III Sum of Squares** | **df** | **Mean Square** | **F** | **Sig.** | **Partial Eta Squared** |
|  | **Corrected Model** | 1.047^a^ | 5 | 0.209 | 14.365 | **<0.001** | 0.253 |
|  | **Intercept** | 0.298 | 1 | 0.298 | 20.448 | **<0.001** | 0.088 |
|  | **Age** | 0.966 | 1 | 0.966 | 66.268 | **<0.001** | 0.238 |
|  | **Sex** | 0.026 | 1 | 0.026 | 1.807 | 0.180 | 0.008 |
|  | **Group** | 0.061 | 3 | 0.02 | 1.404 | 0.243 | 0.019 |
|  | **Error** | 3.089 | 212 | 0.015 |  |  |  |
|  | **Total** | 9.43 | 218 |  |  |  |  |
|  | **Corrected Total** | 4.136 | 217 |  |  |  |  |
|  | a. R Squared = 0.253 (Adjusted R Squared = 0.235) | | | | | | |
| **Serum** | **Corrected Model** | 0.610^b^ | 5 | 0.122 | 10.232 | **<0.001** | 0.246 |
|  | **Intercept** | 0 | 1 | 0 | 0.017 | 0.896 | 0 |
|  | **Age** | 0.558 | 1 | 0.558 | 46.761 | **<0.001** | 0.229 |
|  | **Sex** | 0.032 | 1 | 0.032 | 2.721 | 0.101 | 0.017 |
|  | **Group** | 0.122 | 3 | 0.041 | 3.399 | **0.019** | 0.061 |
|  | **Error** | 1.873 | 157 | 0.012 |  |  |  |
|  | **Total** | 20.031 | 163 |  |  |  |  |
|  | **Corrected Total** | 2.483 | 162 |  |  |  |  |
|  | b. R Squared = 0.246 (Adjusted R Squared = 0.222) | | | | | | |
| **Quotient** | **Corrected Model** | 0.140^c^ | 5 | 0.028 | 1.436 | 0.214 | 0.044 |
|  | **Intercept** | 0.134 | 1 | 0.134 | 6.872 | **0.010** | 0.042 |
|  | **Age** | 0.005 | 1 | 0.005 | 0.262 | 0.610 | 0.002 |
|  | **Sex** | 0.009 | 1 | 0.009 | 0.487 | 0.486 | 0.003 |
|  | **Group** | 0.092 | 3 | 0.031 | 1.573 | 0.198 | 0.029 |
|  | **Error** | 3.04 | 156 | 0.019 |  |  |  |
|  | **Total** | 7.834 | 162 |  |  |  |  |
|  | **Corrected Total** | 3.18 | 161 |  |  |  |  |
|  | c. R Squared = 0.044 (Adjusted R Squared = 0.013) | | | | | | |

Table of ANCOVA analyses (age and sex covariates) with Bonferroni-adjusted post-hoc testing for β2M for the CSF, serum, and quotient. P values are bold for statistically significant results (p < 0.05). CSF = Cerebrospinal Fluid.

Supplementary Table 6. ANCOVA analyses for Haptoglobin

| **ANCOVA Analyses** | | | | | | | |
| --- | --- | --- | --- | --- | --- | --- | --- |
| Dependent Variable: Haptoglobin | | | | | | | |
| **CSF** | **Source** | **Type III Sum of Squares** | **df** | **Mean Square** | **F** | **Sig.** | **Partial Eta Squared** |
|  | **Corrected Model** | 2.784^a^ | 5 | 0.557 | 5.23 | **<0.001** | 0.114 |
|  | **Intercept** | 0.191 | 1 | 0.191 | 1.794 | 0.182 | 0.009 |
|  | **Age** | 0.932 | 1 | 0.932 | 8.757 | **0.003** | 0.041 |
|  | **Sex** | 1.026 | 1 | 1.026 | 9.641 | **0.002** | 0.045 |
|  | **Group** | 0.817 | 3 | 0.272 | 2.557 | 0.056 | 0.036 |
|  | **Error** | 21.719 | 204 | 0.106 |  |  |  |
|  | **Total** | 28.915 | 210 |  |  |  |  |
|  | **Corrected Total** | 24.503 | 209 |  |  |  |  |
|  | a. R Squared = 0.114 (Adjusted R Squared = 0.092) | | | | | | |
| **Serum** | **Corrected Model** | 0.117^b^ | 5 | 0.023 | 0.628 | 0.678 | 0.019 |
|  | **Intercept** | 0.006 | 1 | 0.006 | 0.165 | 0.685 | 0.001 |
|  | **Age** | 0.08 | 1 | 0.08 | 2.133 | 0.146 | 0.013 |
|  | **Sex** | 0.013 | 1 | 0.013 | 0.355 | 0.552 | 0.002 |
|  | **Group** | 0.025 | 3 | 0.008 | 0.224 | 0.879 | 0.004 |
|  | **Error** | 5.937 | 159 | 0.037 |  |  |  |
|  | **Total** | 10.149 | 165 |  |  |  |  |
|  | **Corrected Total** | 6.054 | 164 |  |  |  |  |
|  | b. R Squared = 0.019 (Adjusted R Squared = -0.011) | | | | | | |
| **Quotient** | **Corrected Model** | 0.964^c^ | 5 | 0.193 | 3.028 | 0.013 | 0.1 |
|  | **Intercept** | 0.001 | 1 | 0.001 | 0.02 | 0.888 | 0 |
|  | **Age** | 0.029 | 1 | 0.029 | 0.461 | 0.498 | 0.003 |
|  | **Sex** | 0.563 | 1 | 0.563 | 8.841 | **0.003** | 0.061 |
|  | **Group** | 0.275 | 3 | 0.092 | 1.441 | 0.234 | 0.031 |
|  | **Error** | 8.721 | 137 | 0.064 |  |  |  |
|  | **Total** | 10.229 | 143 |  |  |  |  |
|  | **Corrected Total** | 9.684 | 142 |  |  |  |  |
|  | c. R Squared = 0.100 (Adjusted R Squared = 0.067) | | | | | | |

Table of ANCOVA analyses (age and sex covariates) with Bonferroni-adjusted post-hoc testing for Haptoglobin for the CSF, serum, and quotient. P values are bold for statistically significant results (p < 0.05). CSF = Cerebrospinal Fluid.
