# Supplementary material for: Comparison of inflammatory biomarker levels in neurodegenerative proteinopathies: a case-control study
Source: J Neural Transm (Vienna). 2025 Mar 3;132(6):811–26. doi: 10.1007/s00702-025-02902-6 (PMC12116722; doi:10.1007/s00702-025-02902-6)
Supplement: Supplementary file 2 — Supplementary Material 2 [file 702_2025_2902_MOESM2_ESM.docx]

Online Resource 2

Comparison of inflammatory biomarker levels in neurodegenerative proteinopathies: a case-control study

*Journal of Neural Transmission*

**Sarah E. V. Cook^1, 2 *^, Kateřina Menšíková, Dorota Koníčková, Hedvika Šlanhofová, Kateřina Klíčová, Milan Raška, Jana Zapletalová, David Friedecký, Petr Kaňovský**

^1^Department of Neurology, University Hospital Olomouc, Olomouc, Czech Republic

^2^Department of Neurology, Faculty of Medicine and Dentistry, Palacký University, Olomouc, Czech Republic

^*^ Corresponding Author: Sarah Cook ([sarah.cook01@upol.cz](mailto:sarah.cook01@upol.cz)) (ORCID: 0000-0002-4389-947X)

Supplementary Table 1 Logistic Regression of All Biomarkers for LBD vs. CG

| **Biomarkers** |  | **B** | **S.E.** | **Wald** | **df** | **Sig.** | **Exp(B)** |  |
| --- | --- | --- | --- | --- | --- | --- | --- | --- |
| **C4 Complement** | | -7.290 | 3.346 | 4.746 | 1 | **0.029** | 0.001 | |
| **Haptoglobin** | | 0.546 | 1.199 | 0.207 | 1 | 0.649 | 1.726 | |
| **Transferrin** | | 0.676 | 3.507 | 0.037 | 1 | 0.847 | 1.967 | |
| **C3 Complement** | | 7.085 | 3.132 | 5.119 | 1 | **0.024** | 1193.876 | |
| **Orosomucoid** | | -0.387 | 3.602 | 0.012 | 1 | 0.914 | 0.679 | |
| **β2M** | | -4.276 | 2.700 | 2.507 | 1 | 0.113 | 0.014 | |
| **Serum Transferrin** | | -0.346 | 4.237 | 0.007 | 1 | 0.935 | 0.707 | |
| **Serum Haptoglobin** | | -4.091 | 2.412 | 2.878 | 1 | *0.090* | 0.017 | |
| **Serum Orosomucoid** | | 9.424 | 4.435 | 4.514 | 1 | **0.034** | 12383.590 | |
| **Serum C3 Complement** | | 0.456 | 4.553 | 0.010 | 1 | 0.920 | 1.578 | |
| **Serum C4 Complement** | | 4.747 | 3.535 | 1.803 | 1 | 0.179 | 115.193 | |
| **Serum β2M** | | 0.204 | 2.214 | 0.009 | 1 | 0.927 | 1.226 | |
| **Constant** | | 1.568 | 3.327 | 0.222 | 1 | 0.638 | 4.795 | |

Table of binary logistic regression output of all biomarkers (variables in the equation, full model). “All biomarkers” refers to the combination of all CSF and serum biomarkers. The output of the logistic regression indicates which biomarkers in this combination may have a larger impact on the model, and thus better predictors when determining which group a patient belongs to (LBD vs. control). P values are bold for statistically significant results (p < 0.05). P values are in italics for results trending towards statistical significance (p < 0.1). β2M = β2 microglobulin, CG = Control group, LBD = Lewy Body Disease.

Supplementary Table 2 Logistic Regression of All Biomarkers with Covariates for LBD vs. CG

| **Biomarkers** | **B** | **S.E.** | **Wald** | **df** | **Sig.** | **Exp(B)** |  |
| --- | --- | --- | --- | --- | --- | --- | --- |
| **C4 Complement** | -4.991 | 3.875 | 1.659 | 1 | 0.198 | 0.007 | |
| **Haptoglobin** | 0.283 | 1.368 | 0.043 | 1 | 0.836 | 1.327 | |
| **Transferrin** | -2.410 | 4.441 | 0.295 | 1 | 0.587 | 0.090 | |
| **C3 Complement** | 9.727 | 3.775 | 6.638 | 1 | **0.010** | 16756.645 | |
| **Orosomucoid** | -1.143 | 4.394 | 0.068 | 1 | 0.795 | 0.319 | |
| **β2M** | -1.049 | 3.290 | 0.102 | 1 | 0.750 | 0.350 | |
| **Serum Transferrin** | -3.714 | 4.983 | 0.556 | 1 | 0.456 | 0.024 | |
| **Serum Haptoglobin** | -2.409 | 2.618 | 0.847 | 1 | 0.357 | 0.090 | |
| **Serum Orosomucoid** | 10.212 | 5.333 | 3.667 | 1 | *0.056* | 27240.428 | |
| **Serum C3 Complement** | -0.966 | 5.194 | 0.035 | 1 | 0.852 | 0.381 | |
| **Serum C4 Complement** | 1.300 | 3.919 | 0.110 | 1 | 0.740 | 3.668 | |
| **Serum β2M** | 1.279 | 2.669 | 0.229 | 1 | 0.632 | 3.592 | |
| **Sex** | -0.620 | 0.642 | 0.933 | 1 | 0.334 | 0.538 | |
| **Age** | -0.136 | 0.034 | 15.477 | 1 | **<0.001** | 0.873 | |
| **Constant** | 10.983 | 4.489 | 5.986 | 1 | **0.014** | 58871.056 | |

Table of binary logistic regression output of all biomarkers with covariates (variables in the equation, full model). “All biomarkers” refers to the combination of all CSF and serum biomarkers. The output of the logistic regression indicates which biomarkers in this combination may have a larger impact on the model, and thus better predictors when determining which group a patient belongs to (LBD vs. control). P values are bold for statistically significant results (p < 0.05). P values are in italics for results trending towards statistical significance (p < 0.1). β2M = β2 microglobulin, CG = Control group, LBD = Lewy Body Disease.

Supplementary Table 3 Logistic Regression of CSF Biomarkers for LBD vs. CG

| **Biomarkers** | **B** | **S.E.** | **Wald** | **df** | **Sig.** | **Exp(B)** |  |
| --- | --- | --- | --- | --- | --- | --- | --- |
| **C4 Complement** | 4,468 | 1.921 | 5.406 | 1 | **0.020** | 87.147 | |
| **Haptoglobin** | -0.274 | .631 | 0.189 | 1 | 0.664 | 0.760 | |
| **Transferrin** | 0.335 | 2.125 | 0.025 | 1 | 0.875 | 1.398 | |
| **C3 Complement** | -4.396 | 2.122 | 4.291 | 1 | **0.038** | 0.012 | |
| **Orosomucoid** | -2.219 | 1.798 | 1.523 | 1 | 0.217 | 0.109 | |
| **β2M** | 1.183 | 1.620 | 0.533 | 1 | 0.465 | 3.264 | |
| **Constant** | 2.817 | 2.132 | 1.745 | 1 | 0.187 | 16.719 | |

Table of binary logistic regression output of CSF biomarkers (variables in the equation, full model). The output of the logistic regression indicates which biomarkers in this combination may have a larger impact on the model, and thus better predictors when determining which group a patient belongs to (LBD vs. control). P values are bold for statistically significant results (p < 0.05). β2M = β2 microglobulin, CG = Control group, CSF = Cerebrospinal Fluid, LBD = Lewy Body Disease.

Supplementary Table 4 Logistic Regression of CSF Biomarkers with Covariates for LBD vs. CG

| **Biomarkers** |  | **B** | **S.E.** | **Wald** | **df** | **Sig.** | **Exp(B)** |  |
| --- | --- | --- | --- | --- | --- | --- | --- | --- |
| **C4 Complement** | | 3.364 | 2.075 | 2.629 | 1 | 0.105 | 28.890 | |
| **Haptoglobin** | | -0.322 | 0.721 | 0.200 | 1 | 0.655 | 0.725 | |
| **Transferrin** | | 3.397 | 2.641 | 1.654 | 1 | 0.198 | 29.874 | |
| **C3 Complement** | | -4.995 | 2.530 | 3.897 | 1 | **0.048** | 0.007 | |
| **Orosomucoid** | | -3.084 | 2.045 | 2.274 | 1 | 0.132 | 0.046 | |
| **β2M** | | -3.720 | 2.139 | 3.026 | 1 | *0.082* | 0.024 | |
| **Sex** | | 0.555 | 0.438 | 1.607 | 1 | 0.205 | 1.741 | |
| **Age** | | 0.112 | 0.024 | 22.425 | 1 | **<0.001** | 1.119 | |
| **Constant** | | -6.330 | 3.054 | 4.295 | 1 | **0.038** | 0.002 | |

Table of binary logistic regression output of CSF biomarkers with covariates (variables in the equation, full model). The output of the logistic regression indicates which biomarkers in this combination may have a larger impact on the model, and thus better predictors when determining which group a patient belongs to (LBD vs. control). P values are bold for statistically significant results (p < 0.05). P values are in italics for results trending towards statistical significance (p < 0.1). β2M = β2 microglobulin, CG = Control group, CSF = Cerebrospinal Fluid, LBD = Lewy Body Disease.

Supplementary Table 5 Logistic Regression of Serum Biomarkers for LBD vs. CG

| **Biomarkers** | **B** | **S.E.** | **Wald** | **df** | **Sig.** | **Exp(B)** |  |
| --- | --- | --- | --- | --- | --- | --- | --- |
| **Serum Transferrin** | -4.195 | 2.967 | 1.999 | 1 | 0.157 | 0.015 | |
| **Serum Haptoglobin** | 3.060 | 1.459 | 4.398 | 1 | **0.036** | 21.322 | |
| **Serum Orosomucoid** | -9.299 | 2.617 | 12.625 | 1 | **<0.001** | 0.000 | |
| **Serum C3 Complement** | -5.656 | 3.674 | 2.370 | 1 | 0.124 | 0.003 | |
| **Serum C4 Complement** | -0.383 | 2.205 | 0.030 | 1 | 0.862 | 0.682 | |
| **Serum β2M** | 1.221 | 1.727 | 0.500 | 1 | 0.480 | 3.390 | |
| **Constant** | 0.514 | 1.990 | 0.067 | 1 | 0.796 | 1.672 | |

Table of binary logistic regression output of serum biomarkers (variables in the equation, full model). The output of the logistic regression indicates which biomarkers in this combination may have a larger impact on the model, and thus better predictors when determining which group a patient belongs to (LBD vs. control). P values are bold for statistically significant results (p < 0.05). β2M = β2 microglobulin, CG = Control group, LBD = Lewy Body Disease.

Supplementary Table 6 Logistic Regression of Serum Biomarkers with Covariates for LBD vs. CG

| **Biomarkers** |  | **B** | **S.E.** | **Wald** | **df** | **Sig.** | **Exp(B)** |  |
| --- | --- | --- | --- | --- | --- | --- | --- | --- |
| **Serum Transferrin** | | 0.361 | 3.718 | 0.009 | 1 | 0.923 | 1.435 | |
| **Serum Haptoglobin** | | 1.642 | 1.583 | 1.075 | 1 | 0.300 | 5.165 | |
| **Serum Orosomucoid** | | -9.850 | 3.073 | 10.273 | 1 | **0.001** | 0.000 | |
| **Serum C3 Complement** | | -4.703 | 4.226 | 1.238 | 1 | 0.266 | 0.009 | |
| **Serum C4** | | 1.853 | 2.408 | 0.592 | 1 | 0.442 | 6.380 | |
| **Serum β2M** | | -1.928 | 2.100 | 0.843 | 1 | 0.359 | 0.145 | |
| **Sex** | | 0.910 | 0.521 | 3.049 | 1 | *0.081* | 2.485 | |
| **Age** | | 0.121 | 0.028 | 19.022 | 1 | **<0.001** | 1.129 | |
| **Constant** | | -6.954 | 2.733 | 6.474 | 1 | **0.011** | 0.001 | |

Table of binary logistic regression output of serum biomarkers with covariates (variables in the equation, full model). The output of the logistic regression indicates which biomarkers in this combination may have a larger impact on the model, and thus better predictors when determining which group a patient belongs to (LBD vs. control). P values are bold for statistically significant results (p < 0.05). P values are in italics for results trending towards statistical significance (p < 0.1). β2M = β2 microglobulin, CG = Control group, LBD = Lewy Body Disease.

Supplementary Table 7 Logistic Regression of Significant Biomarkers for LBD vs. CG

| **Biomarkers** | **B** | **S.E.** | **Wald** | **df** | **Sig.** | **Exp(B)** |  |
| --- | --- | --- | --- | --- | --- | --- | --- |
| **Transferrin** | -1.377 | 2.679 | 0.264 | 1 | 0.607 | 0.252 | |
| **Q Transferrin** | 2.903 | 3.218 | 0.814 | 1 | 0.367 | 18.220 | |
| **C3 Complement** | -6.350 | 3.592 | 3.126 | 1 | *0.077* | 0.002 | |
| **Orosomucoid** | -1.037 | 3.133 | 0.110 | 1 | 0.741 | 0.355 | |
| **Q C3 Complement** | 3.390 | 3.174 | 1.141 | 1 | 0.285 | 29.673 | |
| **Serum Orosomucoid** | -5.624 | 3.431 | 2.688 | 1 | 0.101 | 0.004 | |
| **Serum β2M** | 2.394 | 1.802 | 1.765 | 1 | 0.184 | 10.954 | |
| **Constant** | 0.989 | 2.344 | 0.178 | 1 | 0.673 | 2.690 | |

Table of binary logistic regression output of significant biomarkers from ANCOVA (variables in the equation, full model). The output of the logistic regression indicates which biomarkers in this combination may have a larger impact on the model, and thus better predictors when determining which group a patient belongs to (LBD vs. control). P values are in italics for results trending towards statistical significance (p < 0.1). β2M = β2 microglobulin, CG = Control group, LBD = Lewy Body Disease, Q = quotient.

Supplementary Table 8 Logistic Regression of Significant Biomarkers with Covariates for LBD vs. CG

| **Biomarkers** | **B** | **S.E.** | **Wald** | **df** | **Sig.** | **Exp(B)** |  |
| --- | --- | --- | --- | --- | --- | --- | --- |
| **Transferrin** | 3.083 | 3.740 | 0.679 | 1 | 0.410 | 21.815 | |
| **Q Transferrin** | -5.902 | 4.280 | 1.901 | 1 | 0.168 | 0.003 | |
| **C3 Complement** | -4.605 | 4.345 | 1.123 | 1 | 0.289 | 0.010 | |
| **Orosomucoid** | 1.734 | 3.982 | 0.190 | 1 | 0.663 | 5.663 | |
| **Q C3 Complement** | -0.883 | 4.000 | 0.049 | 1 | 0.825 | 0.413 | |
| **Serum Orosomucoid** | -8.206 | 4.534 | 3.275 | 1 | *0.070* | 0.000 | |
| **Serum β2M** | -1.298 | 2.288 | 0.322 | 1 | 0.570 | 0.273 | |
| **Sex** | 0.120 | 0.570 | 0.045 | 1 | 0.833 | 1.128 | |
| **Age** | 0.142 | 0.031 | 21.486 | 1 | **<0.001** | 1.153 | |
| **Constant** | -5.485 | 3.281 | 2.794 | 1 | *0.095* | 0.004 | |

Table of binary logistic regression output of significant biomarkers from ANCOVA with covariates (variables in the equation, full model). The output of the logistic regression indicates which biomarkers in this combination may have a larger impact on the model, and thus better predictors when determining which group a patient belongs to (LBD vs. control). P values are bold for statistically significant results (p < 0.05). P values are in italics for results trending towards statistical significance (p < 0.1). β2M = β2 microglobulin, CG = Control group, LBD = Lewy Body Disease, Q = quotient.

Supplementary Table 9 Logistic Regression of All Biomarkers for MSA vs. CG

| **Biomarkers** | **B** | **S.E.** | **Wald** | **df** | **Sig.** | **Exp(B)** |  |
| --- | --- | --- | --- | --- | --- | --- | --- |
| **C4 Complement** | -3.498 | 5.059 | 0.478 | 1 | 0.489 | 0.030 | |
| **Haptoglobin** | -2.644 | 1.781 | 2.203 | 1 | 0.138 | 0.071 | |
| **Transferrin** | -7.253 | 4.361 | 2.766 | 1 | *0.096* | 0.001 | |
| **C3 Complement** | -15.113 | 6.434 | 5.518 | 1 | **0.019** | 0.000 | |
| **Orosomucoid** | 22.554 | 8.801 | 6.568 | 1 | **0.010** | 6237189162.869 | |
| **β2M** | 10.428 | 4.075 | 6.549 | 1 | **0.010** | 33800.909 | |
| **Serum Transferrin** | 3.363 | 6.154 | 0.299 | 1 | 0.585 | 28.879 | |
| **Serum Haptoglobin** | 8.808 | 3.892 | 5.121 | 1 | **0.024** | 6684.328 | |
| **Serum Orosomucoid** | -27.470 | 10.183 | 7.277 | 1 | **0.007** | 0.000 | |
| **Serum C3 Complement** | 1.329 | 6.267 | 0.045 | 1 | 0.832 | 3.778 | |
| **Serum C4 Complement** | 0.116 | 6.048 | 0.000 | 1 | 0.985 | 1.124 | |
| **Serum β2M** | 0.093 | 2.865 | 0.001 | 1 | 0.974 | 1.098 | |
| **Constant** | -4.740 | 5.337 | 0.789 | 1 | 0.375 | 0.009 | |

Table of binary logistic regression output of all biomarkers (variables in the equation, full model). “All biomarkers” refers to the combination of all CSF and serum biomarkers. The output of the logistic regression indicates which biomarkers in this combination may have a larger impact on the model, and thus better predictors when determining which group a patient belongs to (MSA vs. control). P values are bold for statistically significant results (p < 0.05). P values are in italics for results trending towards statistical significance (p < 0.1). β2M = β2 microglobulin, CG = Control group, MSA = Multiple System Atrophy.

Supplementary Table 10 Logistic Regression of All Biomarkers with Covariates for MSA vs. CG

| **Biomarkers** | **B** | **S.E.** | **Wald** | **df** | **Sig.** | **Exp(B)** |  |
| --- | --- | --- | --- | --- | --- | --- | --- |
| **C4 Complement** | -1.455 | 5.600 | 0.068 | 1 | 0.795 | 0.233 | |
| **Haptoglobin** | -1.664 | 1.998 | 0.694 | 1 | 0.405 | 0.189 | |
| **Transferrin** | -4.567 | 5.860 | 0.607 | 1 | 0.436 | 0.010 | |
| **C3 Complement** | -14.466 | 7.267 | 3.963 | 1 | **0.047** | 0.000 | |
| **Orosomucoid** | 17.048 | 9.367 | 3.312 | 1 | *0.069* | 25348322.115 | |
| **β2M** | 4.574 | 4.648 | 0.969 | 1 | 0.325 | 96.972 | |
| **Serum Transferrin** | 1.207 | 7.813 | 0.024 | 1 | 0.877 | 3.344 | |
| **Serum Haptoglobin** | 7.155 | 4.062 | 3.102 | 1 | *0.078* | 1280.472 | |
| **Serum Orosomucoid** | -21.928 | 10.524 | 4.341 | 1 | **0.037** | 0.000 | |
| **Serum C3 Complement** | 6.498 | 7.557 | 0.739 | 1 | 0.390 | 664.104 | |
| **Serum C4 Complement** | -2.069 | 6.865 | 0.091 | 1 | 0.763 | 0.126 | |
| **Serum β2M** | -0.931 | 3.537 | 0.069 | 1 | 0.792 | 0.394 | |
| **Sex** | 1.425 | 0.984 | 2.095 | 1 | 0.148 | 4.156 | |
| **Age** | 0.138 | 0.058 | 5.657 | 1 | **0.017** | 1.148 | |
| **Constant** | -13.524 | 6.757 | 4.006 | 1 | **0.045** | 0.000 | |

Table of binary logistic regression output of all biomarkers with covariates (variables in the equation, full model). “All biomarkers” refers to the combination of all CSF and serum biomarkers. The output of the logistic regression indicates which biomarkers in this combination may have a larger impact on the model, and thus better predictors when determining which group a patient belongs to (MSA vs. control). P values are bold for statistically significant results (p < 0.05). P values are in italics for results trending towards statistical significance (p < 0.1). β2M = β2 microglobulin, CG = Control group, MSA = Multiple System Atrophy.

Supplementary Table 11 Logistic Regression of CSF Biomarkers for MSA vs. CG

| **Biomarkers** | **B** | **S.E.** | **Wald** | **df** | **Sig.** | **Exp(B)** |  |
| --- | --- | --- | --- | --- | --- | --- | --- |
| **C4 Complement** | -2.546 | 2.290 | 1.236 | 1 | 0.266 | 0.078 | |
| **Haptoglobin** | 0.078 | 1.029 | 0.006 | 1 | 0.940 | 1.081 | |
| **Transferrin** | -1.550 | 2.906 | 0.284 | 1 | 0.594 | 0.212 | |
| **C3 Complement** | -6.521 | 3.865 | 2.847 | 1 | *0.092* | 0.001 | |
| **Orosomucoid** | 2.075 | 3.131 | 0.439 | 1 | 0.508 | 7.961 | |
| **β2M** | 5.312 | 2.543 | 4.363 | 1 | **0.037** | 202.847 | |
| **Constant** | 3.431 | 3.077 | 1.244 | 1 | 0.265 | 30.921 | |

Table of binary logistic regression output of CSF biomarkers (variables in the equation, full model). The output of the logistic regression indicates which biomarkers in this combination may have a larger impact on the model, and thus better predictors when determining which group a patient belongs to (MSA vs. control). P values are bold for statistically significant results (p < 0.05). P values are in italics for results trending towards statistical significance (p < 0.1). β2M = β2 microglobulin, CG = Control group, CSF = Cerebrospinal Fluid, MSA = Multiple System Atrophy.

Supplementary Table 12 Logistic Regression of CSF Biomarkers with Covariates for MSA vs. CG

| **Biomarkers** | **B** | **S.E.** | **Wald** | **df** | **Sig.** | **Exp(B)** |  |
| --- | --- | --- | --- | --- | --- | --- | --- |
| **C4 Complement** | -2.901 | 2.420 | 1.437 | 1 | 0.231 | 0.055 | |
| **Haptoglobin** | 0.594 | 1.231 | 0.233 | 1 | 0.629 | 1.812 | |
| **Transferrin** | -0.622 | 3.748 | 0.028 | 1 | 0.868 | 0.537 | |
| **C3 Complement** | -6.999 | 4.910 | 2.032 | 1 | 0.154 | 0.001 | |
| **Orosomucoid** | 1.045 | 3.819 | 0.075 | 1 | 0.784 | 2.843 | |
| **β2M** | 0.497 | 3.024 | 0.027 | 1 | 0.870 | 1.643 | |
| **Sex** | 1.255 | 0.714 | 3.092 | 1 | *0.079* | 3.508 | |
| **Age** | 0.134 | 0.039 | 11.729 | 1 | **<0.001** | 1.144 | |
| **Constant** | -4.925 | 4.130 | 1.422 | 1 | 0.233 | 0.007 | |

Table of binary logistic regression output of CSF biomarkers with covariates (variables in the equation, full model). The output of the logistic regression indicates which biomarkers in this combination may have a larger impact on the model, and thus better predictors when determining which group a patient belongs to (MSA vs. control). P values are bold for statistically significant results (p < 0.05). P values are in italics for results trending towards statistical significance (p < 0.1). β2M = β2 microglobulin, CG = Control group, CSF = Cerebrospinal Fluid, MSA = Multiple System Atrophy.

Supplementary Table 13 Logistic Regression of Serum Biomarkers for MSA vs. CG

| **Biomarkers** | **B** | **S.E.** | **Wald** | **df** | **Sig.** | **Exp(B)** |  |
| --- | --- | --- | --- | --- | --- | --- | --- |
| **Serum Transferrin** | -7.395 | 4.247 | 3.032 | 1 | *0.082* | 0.001 | |
| **Serum Haptoglobin** | 2.918 | 2.003 | 2.122 | 1 | 0.145 | 18.512 | |
| **Serum Orosomucoid** | -2.624 | 3.780 | 0.482 | 1 | 0.488 | 0.072 | |
| **Serum C3 Complement** | -0.656 | 4.910 | 0.018 | 1 | 0.894 | 0.519 | |
| **Serum C4 Complement** | -4.971 | 3.034 | 2.684 | 1 | 0.101 | 0.007 | |
| **Serum β2M** | 0.036 | 2.284 | 0.000 | 1 | 0.988 | 1.036 | |
| **Constant** | -1.903 | 2.476 | 0.590 | 1 | 0.442 | 0.149 | |

Table of binary logistic regression output of serum biomarkers (variables in the equation, full model). The output of the logistic regression indicates which biomarkers in this combination may have a larger impact on the model, and thus better predictors when determining which group a patient belongs to (MSA vs. control). P values are in italics for results trending towards statistical significance (p < 0.1). β2M = β2 microglobulin, CG = Control group, MSA = Multiple System Atrophy.

Supplementary Table 14 Logistic Regression of Serum Biomarkers with Covariates for MSA vs. CG

| **Biomarkers** | **B** | **S.E.** | **Wald** | **df** | **Sig.** | **Exp(B)** |  |
| --- | --- | --- | --- | --- | --- | --- | --- |
| **Serum Transferrin** | -5.785 | 4.905 | 1.391 | 1 | 0.238 | 0.003 | |
| **Serum Haptoglobin** | 2.895 | 2.131 | 1.847 | 1 | 0.174 | 18.091 | |
| **Serum Orosomucoid** | -6.575 | 4.364 | 2.270 | 1 | 0.132 | 0.001 | |
| **Serum C3 Complement** | 1.906 | 5.252 | 0.132 | 1 | 0.717 | 6.728 | |
| **Serum C4 Complement** | -2.719 | 3.028 | 0.806 | 1 | 0.369 | 0.066 | |
| **Serum β2M** | -1.814 | 2.795 | 0.421 | 1 | 0.516 | 0.163 | |
| **Sex** | 1.604 | 0.774 | 4.299 | 1 | **0.038** | 4.973 | |
| **Age** | 0.122 | 0.044 | 7.842 | 1 | **0.005** | 1.130 | |
| **Constant** | -9.678 | 3.731 | 6.728 | 1 | **0.009** | 0.000 | |

Table of binary logistic regression output of serum biomarkers with covariates (variables in the equation, full model). The output of the logistic regression indicates which biomarkers in this combination may have a larger impact on the model, and thus better predictors when determining which group a patient belongs to (MSA vs. control). P values are bold for statistically significant results (p < 0.05). β2M = β2 microglobulin, CG = Control group, MSA = Multiple System Atrophy.

Supplementary Table 15 Logistic Regression of Significant Biomarkers for MSA vs. CG

| **Biomarkers** | **B** | **S.E.** | **Wald** | **df** | **Sig.** | **Exp(B)** |  |
| --- | --- | --- | --- | --- | --- | --- | --- |
| **Transferrin** | -0.773 | 3.301 | 0.055 | 1 | 0.815 | 0.462 | |
| **Q Transferrin** | -2.909 | 4.468 | 0.424 | 1 | 0.515 | 0.055 | |
| **C3 Complement** | -4.945 | 3.998 | 1.530 | 1 | 0.216 | 0.007 | |
| **Orosomucoid** | 3.807 | 3.140 | 1.470 | 1 | 0.225 | 44.993 | |
| **C4 Complement** | -0.674 | 2.320 | 0.084 | 1 | 0.771 | 0.510 | |
| **Constant** | 2.750 | 3.119 | 0.777 | 1 | 0.378 | 15.647 | |

Table of binary logistic regression output of significant biomarkers from ANCOVA (variables in the equation, full model). The output of the logistic regression indicates which biomarkers in this combination may have a larger impact on the model, and thus better predictors when determining which group a patient belongs to (MSA vs. control). CG = Control group, MSA = Multiple System Atrophy, Q = quotient.

Supplementary Table 16 Logistic Regression of Significant Biomarkers with Covariates for MSA vs. CG

| **Biomarkers** | **B** | **S.E.** | **Wald** | **df** | **Sig.** | **Exp(B)** |  |
| --- | --- | --- | --- | --- | --- | --- | --- |
| **Transferrin** | 3.273 | 4.962 | 0.435 | 1 | 0.509 | 26.390 | |
| **Q Transferrin** | -18.481 | 8.255 | 5.012 | 1 | **0.025** | 0.000 | |
| **C3 Complement** | -1.785 | 6.404 | 0.078 | 1 | 0.780 | 0.168 | |
| **Orosomucoid** | 2.182 | 4.286 | 0.259 | 1 | 0.611 | 8.860 | |
| **C4 Complement** | -0.726 | 2.786 | 0.068 | 1 | 0.794 | 0.484 | |
| **Sex** | 1.532 | 0.872 | 3.086 | 1 | *0.079* | 4.629 | |
| **Age** | 0.229 | 0.071 | 10.522 | 1 | **0.001** | 1.257 | |
| **Constant** | -3.828 | 4.097 | 0.873 | 1 | 0.350 | 0.022 | |

Table of binary logistic regression output of significant biomarkers from ANCOVA with covariates (variables in the equation, full model). The output of the logistic regression indicates which biomarkers in this combination may have a larger impact on the model, and thus better predictors when determining which group a patient belongs to (MSA vs. control). P values are bold for statistically significant results (p < 0.05). P values are in italics for results trending towards statistical significance (p < 0.1). CG = Control group, MSA = Multiple System Atrophy, Q = quotient.

Supplementary Table 17 Logistic Regression of All Biomarkers for 4RT vs. CG

| **Biomarkers** | **B** | **S.E.** | **Wald** | **df** | **Sig.** | **Exp(B)** |  |
| --- | --- | --- | --- | --- | --- | --- | --- |
| **C4 Complement** | 7.254 | 4.806 | 2.278 | 1 | 0.131 | 1413.388 | |
| **Haptoglobin** | -5.540 | 2.112 | 6.881 | 1 | **0.009** | 0.004 | |
| **Transferrin** | -3.593 | 5.113 | 0.494 | 1 | 0.482 | 0.028 | |
| **C3 Complement** | -11.223 | 5.728 | 3.839 | 1 | *0.050* | 0.000 | |
| **Orosomucoid** | 14.674 | 7.520 | 3.808 | 1 | *0.051* | 2359352.948 | |
| **β2M** | 3.074 | 3.739 | 0.676 | 1 | 0.411 | 21.635 | |
| **Serum β2M** | -2.723 | 3.596 | 0.573 | 1 | 0.449 | 0.066 | |
| **Serum Transferrin** | 4.127 | 7.153 | 0.333 | 1 | 0.564 | 61.990 | |
| **Serum Haptoglobin** | 9.676 | 4.487 | 4.650 | 1 | **0.031** | 15933.470 | |
| **Serum Orosomucoid** | -14.141 | 8.684 | 2.652 | 1 | 0.103 | 0.000 | |
| **Serum C3 Complement** | 25.363 | 10.021 | 6.405 | 1 | **0.011** | 103512447257.692 | |
| **Serum C4 Complement** | -6.413 | 5.362 | 1.431 | 1 | 0.232 | 0.002 | |
| **Constant** | -14.627 | 5.940 | 6.064 | 1 | **0.014** | 0.000 | |

Table of binary logistic regression output of all biomarkers (variables in the equation, full model). “All biomarkers” refers to the combination of all CSF and serum biomarkers. The output of the logistic regression indicates which biomarkers in this combination may have a larger impact on the model, and thus better predictors when determining which group a patient belongs to (4RT vs. control). P values are bold for statistically significant results (p < 0.05). P values are in italics for results trending towards statistical significance (p < 0.1). β2M = β2 microglobulin, CG = Control group, 4RT = 4-Repeat Tauopathy.

Supplementary Table 18 Logistic Regression of All Biomarkers with Covariates for 4RT vs. CG

| **Biomarkers** | **B** | **S.E.** | **Wald** | **df** | **Sig.** | **Exp(B)** |  |
| --- | --- | --- | --- | --- | --- | --- | --- |
| **C4 Complement** | 8.504 | 6.439 | 1.744 | 1 | 0.187 | 4934.472 | |
| **Haptoglobin** | -5.849 | 2.745 | 4.539 | 1 | **0.033** | 0.003 | |
| **Transferrin** | 0.256 | 8.187 | 0.001 | 1 | 0.975 | 1.292 | |
| **C3 Complement** | -11.272 | 7.078 | 2.536 | 1 | 0.111 | 0.000 | |
| **Orosomucoid** | 9.589 | 9.563 | 1.005 | 1 | 0.316 | 14602.161 | |
| **β2M** | -5.462 | 5.666 | 0.929 | 1 | 0.335 | 0.004 | |
| **Serum β2M** | -6.392 | 5.292 | 1.459 | 1 | 0.227 | 0.002 | |
| **Serum Transferrin** | 3.275 | 9.762 | 0.113 | 1 | 0.737 | 26.447 | |
| **Serum Haptoglobin** | 10.410 | 5.483 | 3.604 | 1 | *0.058* | 33178.581 | |
| **Serum Orosomucoid** | -6.457 | 11.037 | 0.342 | 1 | 0.559 | 0.002 | |
| **Serum C3 Complement** | 25.827 | 11.918 | 4.696 | 1 | **0.030** | 164601465021.188 | |
| **Serum C4 Complement** | -7.229 | 6.643 | 1.184 | 1 | 0.277 | 0.001 | |
| **Sex** | -0.239 | 1.237 | 0.037 | 1 | 0.847 | 0.787 | |
| **Age** | 0.209 | 0.084 | 6.151 | 1 | **0.013** | 1.232 | |
| **Constant** | -26.403 | 9.107 | 8.405 | 1 | **0.004** | 0.000 | |

Table of binary logistic regression output of all biomarkers with covariates (variables in the equation, full model). “All biomarkers” refers to the combination of all CSF and serum biomarkers. The output of the logistic regression indicates which biomarkers in this combination may have a larger impact on the model, and thus better predictors when determining which group a patient belongs to (4RT vs. control). P values are bold for statistically significant results (p < 0.05). P values are in italics for results trending towards statistical significance (p < 0.1). β2M = β2 microglobulin, CG = Control group, 4RT = 4-Repeat Tauopathy.

Supplementary Table 19 Logistic Regression of CSF Biomarkers for 4RT vs. CG

| **Biomarkers** | **B** | **S.E.** | **Wald** | **df** | **Sig.** | **Exp(B)** |  |
| --- | --- | --- | --- | --- | --- | --- | --- |
| **C4 Complement** | 0.876 | 2.006 | 0.191 | 1 | 0.662 | 2.402 | |
| **Haptoglobin** | -1.083 | 0.863 | 1.575 | 1 | 0.209 | 0.338 | |
| **Transferrin** | 1.936 | 2.784 | 0.484 | 1 | 0.487 | 6.933 | |
| **C3 Complement** | -7.929 | 3.214 | 6.087 | 1 | **0.014** | 0.000 | |
| **Orosomucoid** | 6.039 | 2.614 | 5.337 | 1 | **0.021** | 419.314 | |
| **β2M** | 2.821 | 2.043 | 1.907 | 1 | 0.167 | 16.797 | |
| **Constant** | -3.673 | 3.007 | 1.492 | 1 | 0.222 | 0.025 | |

Table of binary logistic regression output of CSF biomarkers (variables in the equation, full model). The output of the logistic regression indicates which biomarkers in this combination may have a larger impact on the model, and thus better predictors when determining which group a patient belongs to (4RT vs. control). P values are bold for statistically significant results (p < 0.05). β2M = β2 microglobulin, CG = Control group, CSF = Cerebrospinal Fluid, 4RT = 4-Repeat Tauopathy.

Supplementary Table 20 Logistic Regression of CSF Biomarkers with Covariates for 4RT vs. CG

| **Biomarkers** | **B** | **S.E.** | **Wald** | **df** | **Sig.** | **Exp(B)** |  |
| --- | --- | --- | --- | --- | --- | --- | --- |
| **C4 Complement** | 0.209 | 2.126 | 0.010 | 1 | 0.922 | 1.233 | |
| **Haptoglobin** | -0.600 | 0.952 | 0.398 | 1 | 0.528 | 0.549 | |
| **Transferrin** | 2.582 | 3.194 | 0.653 | 1 | 0.419 | 13.227 | |
| **C3 Complement** | -8.313 | 3.687 | 5.085 | 1 | **0.024** | 0.000 | |
| **Orosomucoid** | 4.289 | 2.849 | 2.266 | 1 | 0.132 | 72.863 | |
| **β2M** | 0.422 | 2.343 | 0.032 | 1 | 0.857 | 1.526 | |
| **Sex** | 0.528 | 0.552 | 0.914 | 1 | 0.339 | 1.695 | |
| **Age** | 0.115 | 0.033 | 12.337 | 1 | **<0.001** | 1.122 | |
| **Constant** | -9.685 | 3.870 | 6.262 | 1 | **0.012** | 0.000 | |

Table of binary logistic regression output of CSF biomarkers with covariates (variables in the equation, full model). The output of the logistic regression indicates which biomarkers in this combination may have a larger impact on the model, and thus better predictors when determining which group a patient belongs to (4RT vs. control). P values are bold for statistically significant results (p < 0.05). β2M = β2 microglobulin, CG = Control group, CSF = Cerebrospinal Fluid, 4RT = 4-Repeat Tauopathy.

Supplementary Table 21 Logistic Regression of Serum Biomarkers for 4RT vs. CG

| **Biomarkers** | **B** | **S.E.** | **Wald** | **df** | **Sig.** | **Exp(B)** |  |
| --- | --- | --- | --- | --- | --- | --- | --- |
| **Serum Transferrin** | -1.079 | 5.203 | 0.043 | 1 | 0.836 | 0.340 | |
| **Serum Haptoglobin** | 1.101 | 2.134 | 0.266 | 1 | 0.606 | 3.008 | |
| **Serum Orosomucoid** | 0.354 | 3.854 | 0.008 | 1 | 0.927 | 1.425 | |
| **Serum C3 Complement** | 11.705 | 5.918 | 3.912 | 1 | **0.048** | 121153.722 | |
| **Serum C4 Complement** | -2.948 | 3.326 | 0.786 | 1 | 0.375 | 0.052 | |
| **Serum β2M** | -2.765 | 2.562 | 1.165 | 1 | 0.281 | 0.063 | |
| **Constant** | -4.379 | 3.013 | 2.113 | 1 | 0.146 | 0.013 | |

Table of binary logistic regression output of serum biomarkers (variables in the equation, full model). The output of the logistic regression indicates which biomarkers in this combination may have a larger impact on the model, and thus better predictors when determining which group a patient belongs to (4RT vs. control). P values are bold for statistically significant results (p < 0.05). β2M = β2 microglobulin, CG = Control group, 4RT = 4-Repeat Tauopathy.

Supplementary Table 22 Logistic Regression of Serum Biomarkers with Covariates for 4RT vs. CG

| **Biomarkers** | **B** | **S.E.** | **Wald** | **df** | **Sig.** | **Exp(B)** | |
| --- | --- | --- | --- | --- | --- | --- | --- |
| **Serum Transferrin** | 1.970 | 6.505 | 0.092 | 1 | 0.762 | 7.167 |  |
| **Serum Haptoglobin** | 0.066 | 2.516 | 0.001 | 1 | 0.979 | 1.068 |  |
| **Serum Orosomucoid** | -0.605 | 4.885 | 0.015 | 1 | 0.901 | 0.546 |  |
| **Serum C3 Complement** | 15.809 | 8.168 | 3.746 | 1 | *0.053* | 7342672.561 |  |
| **Serum C4 Complement** | 0.823 | 3.698 | 0.050 | 1 | 0.824 | 2.277 |  |
| **Serum β2M** | -8.830 | 3.837 | 5.296 | 1 | **0.021** | 0.000 |  |
| **Sex** | 0.509 | 0.876 | 0.337 | 1 | 0.562 | 1.663 |  |
| **Age** | 0.188 | 0.057 | 10.763 | 1 | **0.001** | 1.207 |  |
| **Constant** | -14.435 | 4.583 | 9.919 | 1 | **0.002** | 0.000 |  |

Table of binary logistic regression output of serum biomarkers with covariates (variables in the equation, full model). The output of the logistic regression indicates which biomarkers in this combination may have a larger impact on the model, and thus better predictors when determining which group a patient belongs to (4RT vs. control). P values are bold for statistically significant results (p < 0.05). P values are in italics for results trending towards statistical significance (p < 0.1). β2M = β2 microglobulin, CG = Control group, 4RT = 4-Repeat Tauopathy.

Supplementary Table 23 Logistic Regression outputs for interpretation of models for the combinations of Biomarkers

| **Group** | **Combinations** | **Chi-Squared** | **df** | **Sig.** | **Nagelkerke R^2^** | **Overall Correctly Predicted (%)** |
| --- | --- | --- | --- | --- | --- | --- |
| **LBD vs. CG** | All Biomarkers | 38.422 | 12 | **<0.001** | 0.385 (38.5%) | 72.6% |
|  | All Biomarkers with covariates | 61.072 | 14 | **<0.001** | 0.558 (55.8%) | 76.1% |
|  | CSF Biomarkers | 23.534 | 6 | **<0.001** | 0.196 (19.6%) | 64.2% |
|  | CSF Biomarkers with covariates | 55.430 | 8 | **<0.001** | 0.417 (41.7%) | 74.3% |
|  | Serum Biomarkers | 25.559 | 6 | **<0.001** | 0.250 (25.0%) | 69.4% |
|  | Serum Biomarkers with covariates | 55.294 | 8 | **<0.001** | 0.482 (48.2%) | 75.0% |
|  | Significant Biomarkers | 26.157 | 7 | **<0.001** | 0.263 (26.3%) | 68.3% |
|  | Significant Biomarkers with covariates | 59.185 | 9 | **<0.001** | 0.522 (52.2%) | 79.2% |
| **MSA vs. CG** | All Biomarkers | 23.055 | 12 | **0.027** | 0.394 (39.4%) | 84.6% |
|  | All Biomarkers with covariates | 33.769 | 14 | **0.002** | 0.541 (54.1%) | 85.9% |
|  | CSF Biomarkers | 17.682 | 6 | **0.007** | 0.255 (25.5%) | 82.3% |
|  | CSF Biomarkers with covariates | 38.765 | 8 | **<0.001** | 0.504 (50.4%) | 87.5% |
|  | Serum Biomarkers | 9.182 | 6 | 0.164 | 0.160 (16.0%) | 79.3% |
|  | Serum Biomarkers with covariates | 23.754 | 8 | **0.003** | 0.381 (38.1%) | 80.5% |
|  | Significant Biomarkers | 10.375 | 5 | *0.065* | 0.180 (18.0%) | 79.1% |
|  | Significant Biomarkers with covariates | 38.899 | 7 | **<0.001** | 0.578 (57.8%) | 87.2% |
| **4RT vs. CG** | All Biomarkers | 22.089 | 12 | **0.037** | 0.426 (42.6%) | 86.5% |
|  | All Biomarkers with covariates | 34.419 | 14 | **0.002** | 0.615 (61.5%) | 90.5% |
|  | CSF Biomarkers | 11.129 | 6 | *0.084* | 0.150 (15.0%) | 71.6% |
|  | CSF Biomarkers with covariates | 29.056 | 8 | **<0.001** | 0.359 (35.9%) | 75.5% |
|  | Serum Biomarkers | 6.860 | 6 | 0.334 | 0.132 (13.2%) | 83.3% |
|  | Serum Biomarkers with covariates | 28.074 | 8 | **<0.001** | 0.478 (47.8%) | 89.3% |

Table of binary logistic regression outputs for interpretation of the models for the combinations of all biomarkers, CSF biomarkers, serum biomarkers and significant biomarkers from the ANCOVA, with and without covariates. “All biomarkers” refers to the combination of all CSF and serum biomarkers. The Sig. column identifies whether or not the overall model was statistically significant when compared to the null model. Nagelkerke R^2^ explains how much variation is explained by the full model. Overall Correctly Predicted (%) identifies the percentage of individuals that were correctly classified/predicted according to the full model. Therefore, this table shows which combinations of biomarkers may produce a better model for correctly predicted whether a patient is a part of the disease group (LBD, MSA, 4RT) or CG. P values are bold for statistically significant results (p < 0.05). P values are in italics for results trending towards statistical significance (p < 0.1). CG = Control group, LBD = Lewy Body Disease, MSA = Multiple System Atrophy, 4RT = 4-Repeat Tauopathy.


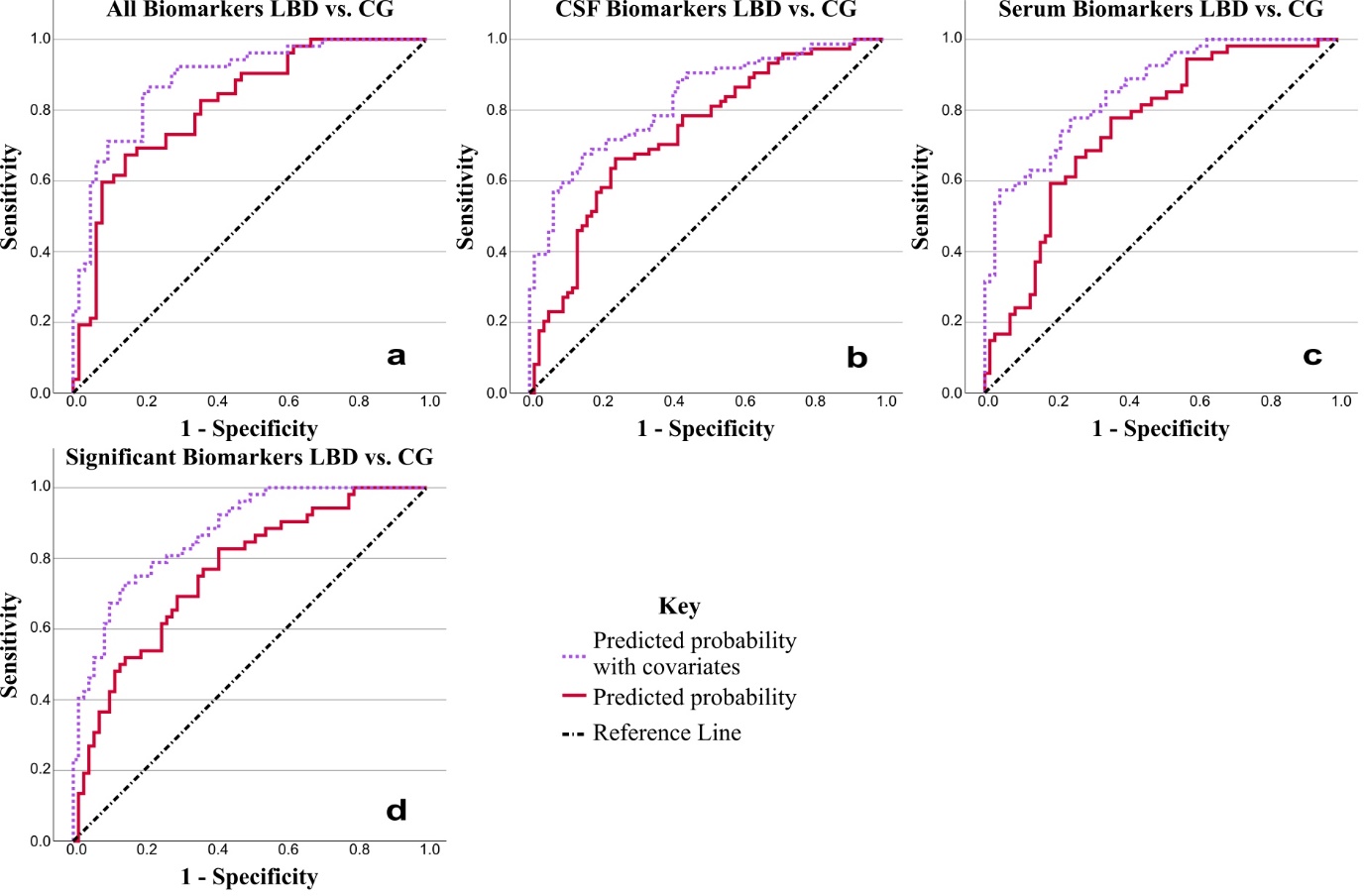


**Supplementary Fig. 1** ROC curves for the combinations of all biomarkers (a), CSF biomarkers (b), serum biomarkers (c) and significant biomarkers from the ANCOVA (d) in LBD vs. CG. “All biomarkers” refers to the combination of all CSF and serum biomarkers. The solid red line is the ROC curve created using the predicted probabilities produced from the combination of variables (biomarker data only) in binary logistic regression, and the dotted purple line is the ROC curve created using the predicted probabilities produced from the combination of variables with covariates (biomarker data, sex and age) in binary logistic regression. The straight diagonal dash and dot black line is the reference line. In all parts, predicted probability curves, with and without covariates, are to the left of the reference line, with covariates producing a better model. See Online Resource 2, Supplementary Table 24 (below) for areas under the curve. Positive actual state = LBD. CG = Control Group, LBD = Lewy Body Disease.


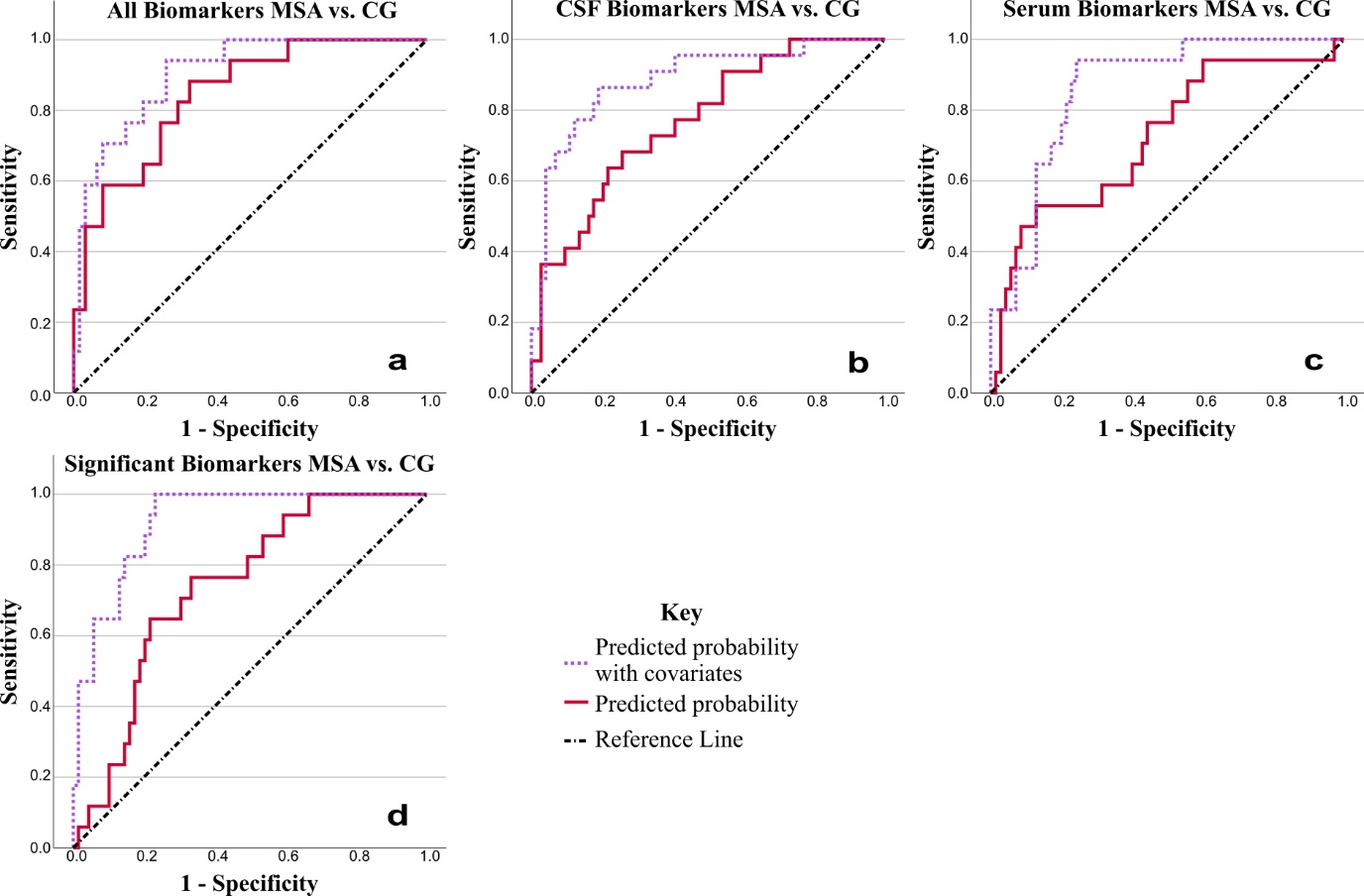


**Supplementary** **Fig. 2** ROC curves for the combinations of all biomarkers (a), CSF biomarkers (b), serum biomarkers (c) and significant biomarkers from the ANCOVA (d) in MSA vs. CG. “All biomarkers” refers to the combination of all CSF and serum biomarkers. The solid red line is the ROC curve created using the predicted probabilities produced from the combination of variables (biomarker data only) in binary logistic regression, and the dotted purple line is the ROC curve created using the predicted probabilities produced from the combination of variables with covariates (biomarker data, sex and age) in binary logistic regression. The straight diagonal dash and dot black line is the reference line. In all parts, predicted probability curves, with and without covariates, are to the left of the reference line, with covariates producing a better model. See Online Resource 2, Supplementary Table 24 (below) for areas under the curve. Positive actual state = MSA. CG = Control Group, MSA = Multiple System Atrophy.


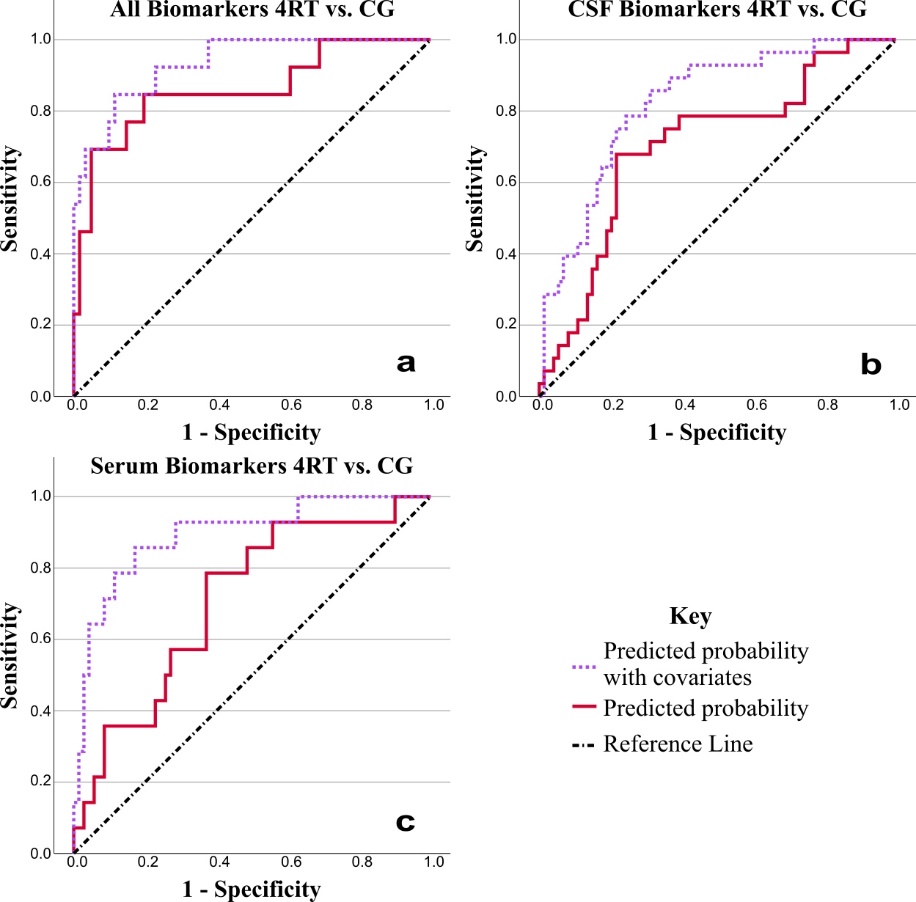


**Supplementary Fig.** **3** ROC curves for the combinations of all biomarkers (a), CSF biomarkers (b) and serum biomarkers (c) in 4RT vs. CG. “All biomarkers” refers to the combination of all CSF and serum biomarkers. The solid red line is the ROC curve created using the predicted probabilities produced from the combination of variables (biomarker data only) in binary logistic regression, and the dotted purple line is the ROC curve created using the predicted probabilities produced from the combination of variables with covariates (biomarker data, sex and age) in binary logistic regression. The straight diagonal dash and dot black line is the reference line. In all parts, predicted probability curves, with and without covariates, are to the left of the reference line, with covariates producing a better model. See Online Resource 2, Supplementary Table 24 (below) for areas under the curve. Positive actual state = 4RT. CG = Control Group, 4RT = 4-Repeat Tauopathy.

Supplementary Table 24 ROC curve analysis parameters

| Test Result Variables | | Area Under the Curve | Standard Error | Asymptotic  P Value | Asymptotic 95% Confidence Interval | |
| --- | --- | --- | --- | --- | --- | --- |
|  |  |  |  |  | Lower Bound | Upper Bound |
| LBD vs. CG | All Biomarkers | 0.817 | 0.040 | **<0.001** | 0.739 | 0.895 |
|  | All Biomarkers with covariates | 0.887 | 0.031 | **<0.001** | 0.836 | 0.947 |
|  | CSF Biomarkers | 0.734 | 0.041 | **<0.001** | 0.653 | 0.814 |
|  | CSF Biomarkers with covariates | 0.826 | 0.033 | **<0.001** | 0.760 | 0.891 |
|  | Serum Biomarkers | 0.751 | 0.044 | **<0.001** | 0.665 | 0.836 |
|  | Serum Biomarkers with covariates | 0.857 | 0.032 | **<0.001** | 0.793 | 0.920 |
|  | Significant Biomarkers | 0.760 | 0.043 | **<0.001** | 0.675 | 0.846 |
|  | Significant Biomarkers with covariates | 0.872 | 0.031 | **<0.001** | 0.812 | 0.933 |
| MSA vs. CG | All Biomarkers | 0.844 | 0.050 | **<0.001** | 0.745 | 0.942 |
|  | All Biomarkers with covariates | 0.905 | 0.036 | **<0.001** | 0.834 | 0.977 |
|  | CSF Biomarkers | 0.769 | 0.056 | **<0.001** | 0.660 | 0.878 |
|  | CSF Biomarkers with covariates | 0.885 | 0.043 | **<0.001** | 0.801 | 0.968 |
|  | Serum Biomarkers | 0.723 | 0.072 | **0.002** | 0.582 | 0.864 |
|  | Serum Biomarkers with covariates | 0.860 | 0.043 | **<0.001** | 0.776 | 0.943 |
|  | Significant Biomarkers | 0.738 | 0.059 | **<0.001** | 0.622 | 0.854 |
|  | Significant Biomarkers with covariates | 0.923 | 0.028 | **<0.001** | 0.867 | 0.979 |
| 4RT vs. CG | All Biomarkers | 0.859 | 0.065 | **<0.001** | 0.731 | 0.987 |
|  | All Biomarkers with covariates | 0.933 | 0.035 | **<0.001** | 0.865 | 1.001 |
|  | CSF Biomarkers | 0.704 | 0.058 | **<0.001** | 0.589 | 0.818 |
|  | CSF Biomarkers with covariates | 0.823 | 0.044 | **<0.001** | 0.736 | 0.909 |
|  | Serum Biomarkers | 0.709 | 0.072 | **<0.001** | 0.567 | 0.851 |
|  | Serum Biomarkers with covariates | 0.894 | 0.048 | **<0.001** | 0.800 | 0.988 |

The area under the curve, standard error, asymptotic P value, and asymptotic 95% Confidence Intervals of each ROC curve from Supplementary Figs 1-3 (predicted probabilities of the biomarkers (all, CSF, serum, or significant biomarkers from the ANCOVA), with and without covariates). P values are shown in bold for statistically significant results (p < 0.05). CG = Control Group, CSF = Cerebrospinal Fluid, LBD = Lewy Body Disease, MSA = Multiple System Atrophy, 4RT = 4-Repeat Tauopathy.
